# Research on the evaluation and spatial layout of high quality development of higher education in China

**DOI:** 10.1371/journal.pone.0347744

**Published:** 2026-07-09

**Authors:** Wen Li, Jinfa Shi, Qiting Zuo, Jianqin Ma, Meng Qiu, Yushun Zhang

**Affiliations:** 1 North China University of Water Resources and Electricity, Zhengzhou, China; 2 School of Water Conservancy Engineering, Zhengzhou University, Zhengzho, China; 3 Yellow River Institute for Ecological Protection & Regional Coordination Development, Zhengzho, China; 4 Law School, Henan University of Economics and Law, Zhengzhou, China; 5 Institute of Water Resources and Rural Water Conservancy, Henan Water Conservancy Science and Technology Application Center, Zhengzhou, China; Northeast Normal University, CHINA

## Abstract

As a social subsystem, the layout of higher education is related to the degree of harmony in the economy and society. Based on the panel data from the “China Statistical Yearbook”, “China Education Statistical Yearbook” and the “Education Statistical Yearbook” of China’s provincial administrative regions from 2000 to 2022, considering factors such as sustainable economic and social development capacity, regional scientific and technological cultural development level, and spatial layout of higher education, this study determines the degree of harmonic development among the three subsystems. This paper constructs an indicator system for harmonious development of China’s higher education, and uses the method of “single indicator quantification-multi indicator synthesis-multi criteria integration” (SMI-P) to determine the harmonious development index of China’s higher education. The ArcGIS spatial analysis method is used to study the spatial distribution changes of higher education harmonious development in China’s 31 provincial administrative regions. Then, the Tobit model is used to analyze the impact of various economic and social factors, scientific and cultural factors or layout of higher education factors on the harmonious development of higher education, in order to optimize the spatial layout of China’s higher education resources, balance education development, narrow regional disparities, promote regional coordination, and enhance the overall competitiveness of higher education. Research has found that: (1) The development of higher education subsystems shows a trend of convergence; (2) Over the past 23 years (from 2000 to 2023), the spatial centroid of harmonious development of higher education (HDHE) in China shifted southward; (3) There is a strong spatial interaction between 3 subsystems of harmonious development of higher education in China(economic society subsystem, technology and culture subsystem, and higher education subsystem); (4) The main factors affecting HDHE in China include the degree of regional ecological civilization, the ratio of university students to teachers and the per capita domestic patent application authorization.

## 1. Introduction

The harmonious development of higher education (HDHE) is a necessary condition for promoting regional education and overall regional development, and has important practical significance for promoting the construction of a harmonious regional society [1]. The fourth item of the United Nations SDGs states that “high-quality education” refers to ensuring inclusive and fair quality education, promoting lifelong learning opportunities for all. Inclusive and fair high-quality education requires a balanced spatial layout of higher education (SLHE) to support it. The SLHE is an important factor affecting HDHE. SLHE refers to the distribution of the number, structure, level, strength and other constituent elements of higher education in a certain geographical or administrative region. Its essence is the relationship between higher education and economic, social, technological, and cultural development within a certain time and space. It is meaningless to explore the SLHE without economic and social development [2].

The SLHE is superficially a concept of educational geography, but essentially a comprehensive reflection of natural endowment and acquired development, educational foundation and economic and social development level, as well as national unified layout and local differential development [3]. The causes of SLHE are complex, including political factors as well as economic, demographic, geographical, historical, and cultural factors [4–6]. SLHE is a prerequisite for HDHE, which is also an important engine of economic growth. The economic growth of a region is closely related to the scale and quality of higher education. The expansion of investment in higher education, the improvement of quality, and the improvement of efficiency all promote economic growth to varying degrees [7]. SLHE in a region not only provides talent capital and intellectual support for the economic growth of the region, but also promotes the dissemination of knowledge production, technological innovation, and technological progress through the teaching and research of universities, promoting the construction and development of regional cultural industries and social service undertakings. At the same time, the growth of regional economy can provide material basis, financial guarantee, and software support for the investment in higher education in the region, provide impetus for the structural transformation and upgrading of higher education, promote the development of regional science and technology culture, and thus form a virtuous interactive cycle [8]. Understanding the relationship between SLHE and the development of regional economic society, technology and culture, as well as how to establish a positive interactive relationship between these three, is of great significance for deepening the reform and development of the higher education field, improving the competitiveness of the regional economy, and achieving sustainable development of the regional economy.

For a long time, the issue of HDHE and the coordinated development of regional economic, social, technological, and cultural factors has been the focus of attention for scholars. There are analyses examines institutional building and sustainable development of higher education in transition economies [9]. At a time when countries seek to redefine their education policies towards stabilization, prosperity, and democratization of opportunities, the idea of community college offers a powerful connective solution to community, industry, and national economies [10]. By taking stock of research on the school-to-HE transition [11] contribute to broaden the debate on the topic and reflects upon the relation between education and economic society. With the continuous changes and development of the spatial layout of higher education, higher requirements have been put forward for the sustainable development of regional economy. To confirm the close relationship between the spatial layout of higher education and the interaction between regional economy and society, scholars have attempted to analyze its correlation from different perspectives. Strategic learning, as a dynamic capability rooted in Senge’s learning organization theory, not only fosters continuous adaptation but also underpins the internationalization of higher education. Internationalization drives cross-border academic exchanges and resource integration, while learning organization enhances internal innovation capacity; R&D activities in universities provide businesses a competitive edge by bridging academia-industry gaps, forming a complete logical chain among internationalization, learning organization, R&D and competitive edge. Similarly, regional innovation efforts—such as those measured by synthetic quality-of-life indicators in urban/rural areas—attract FDI, which in turn amplifies the outcomes of educational innovation. Regional innovation capacity favors the absorption of foreign direct investment, and further realizes beneficial innovation outcomes through knowledge spillover and technology transfer, which has been widely practiced in the European Union, Spain, Germany and other developed economies. These linkages highlight the multifaceted role of higher education in driving sustainable development. [12]. With social distancing being in place and the increased use of technology [13] present opportunities for universities in emerging countries to meet the growing demand of prospective students who may not be able to travel abroad for their higher education, opportunities to improve the quality of education and build a partnership, albeit virtually with different Universities around the world.

Among the three goals of regional coordinated development proposed by the CPC in 2017, the harmonious development of basic public services is the primary goal. The coordinated development of higher education spatial layout and the realization of regional harmony in educational resources is one of the urgent problems to be solved in comprehensively improving the quality of talent cultivation. After China successfully achieved goal one of poverty alleviation in 2020, educational harmony and high-quality education have become important reform directions [14]. Integrating higher education with the sustainable development of technology and culture, as well as the development of economic society, and taking corresponding actions are the development requirements of SDGs and the development policy of the Chinese government. In response to the new situation and requirements of regional higher education investment and economic growth, how to promote the *spatial layout of higher education* (SLHE) through promotion of technological and cultural development and utilization of regional economic growth achievements is a very noteworthy issue. The 6E evaluation index system is constructed to measure the sustainable higher education development of the 31 provincial regions in China, utilizing the information entropy weight-TOPSIS method, with comprehensive dimensions including economy, effectiveness, and equality [15]. More specifically, discussion of the higher education development trends in China went through the massification, diversification and internationalisation [16]. AHP-NBM Fuzzy Comprehensive Evaluation Model (ANFCE) was adopt to select the health levels of 7 countries for calculation and analysis, and evaluated the stability of the model [17].

In summary, there are still some shortcomings that need to be supplemented and improved in the research on the interaction between the spatial layout of higher education and economic and social development. Firstly, in existing research, the spatial layout of higher education and regional economic and social development issues are often separately considered as research objects, while neglecting the overall study of the interaction between higher education and regional economic and social development. Secondly, there is a lack of research on the correlation between China’s overall higher education and regional economic and social development, especially the lack of research that covers both higher education, technology and culture, and economic and social development. Thirdly, existing research mainly focuses on qualitative research on the relationship between higher education and economic and social development. From a quantitative perspective, it mostly evaluates the coupling and coordination between higher education and economic and social development systems, and rarely involves interaction mechanisms in terms of content.

While prior studies have extensively analyzed SLHE’s historical evolution [18] and policy optimization [19], this study contributes to the literature in three ways: (a) introducing a novel SMI-P-ArcGIS integrated framework to quantify HDHE’s spatial-temporal dynamics at provincial level, filling the gap in micro-scale empirical evidence; (b) identifying ‘ecological civilization’ as a key factor overlooked in existing SLHE models; and (c) proposing differentiated policy pathways for eastern-coastal vs. western-inland regions based on Tobit regression results, advancing the discourse on spatial justice in higher education.

The positive interaction between higher education and regional economy is also affected by internationalization and R&D. The absorption of foreign direct investment promotes knowledge spillover and technological progress, which is consistent with the innovation-driven development models implemented in regions such as the European Union, Spain, and Germany.

Internationalization (e.g., FDI absorption) moderates the relationship between R&D investment and HDHE, as seen in Eastern China’s innovation hubs like Shanghai and Guangdong, where FDI inflows have accelerated technology transfer. This aligns with global patterns, such as Catalonia’s RIS3 strategy in Spain or Bavaria’s High-Tech Offensive in Germany, where FDI-driven innovation synergies are prioritized.

The remaining structure of this article is as follows: Chapter 2 briefly introduces the overview of the research area, as well as the collection and processing of data, and elaborates on the methods used in this article. Chapter 3 provides a comprehensive analysis of the calculation results from multiple dimensions, delving into the correlation and interaction between economic society, technology and culture, and higher education. The main conclusion and shortcomings of this article are presented in Chapter 4.

## 2. Materials and methods

### 2.1. Overview of the research area

The change in the unbalanced spatial layout of higher education is constrained by the level of economic development and population quality [20].

The spatial balance of higher education is superficially an educational issue, but essentially an economic issue. Multiple factors of high education intertwined and influencing each other have become hot and difficult issues that affect the overall economic and social development [21].

The definition of SLHE refers to the distribution of higher education institutions’ quantity, structure, and hierarchy across geographical/administrative regions, and their dynamic interactions with regional socioeconomic development. This differs from ‘balanced development’ (equity-focused) or ‘resource optimization’ (efficiency-focused), as SLHE emphasizes spatial-temporal coordination among multiple subsystems (economic, technological, educational).

As for Conceptual Boundaries, which means SLHE is distinct from—but interdependent with—three related concepts: (i) HDHE measures outcome harmony among subsystems; (ii) regional equity evaluates access fairness; (iii) resource allocation focuses on input efficiency. Our study bridges these concepts by examining how SLHE shapes HDHE through spatial econometrics.

**Fig 1:**
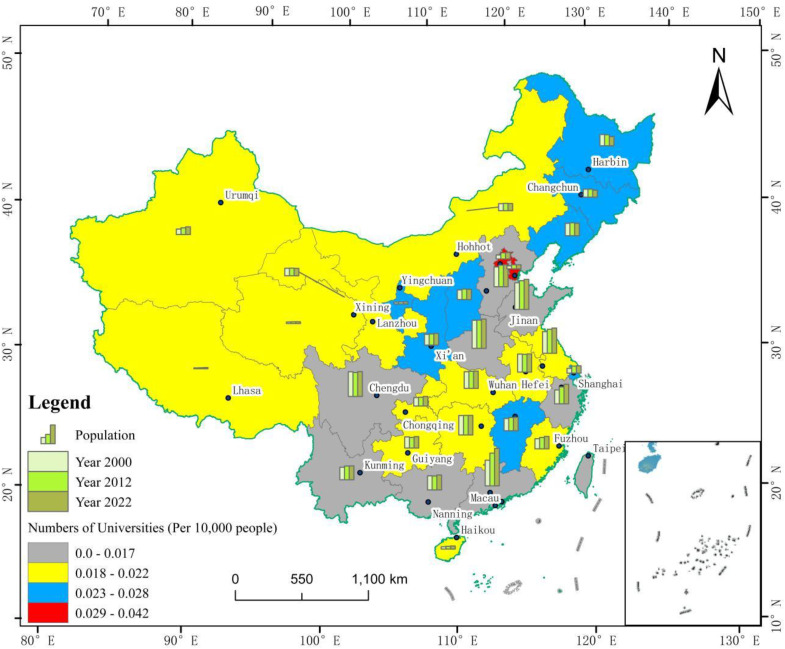
Spatial Distribution Map of China’s Economic and Population Development. *Note:* The administrative boundary data of Chinese provinces (ADM1 level) were downloaded from the geoBoundaries Global Database of Political Administrative Boundaries (https://www.geoboundaries.org/). This dataset is made available under the Creative Commons Attribution 4.0 International License (CC BY 4.0), which permits unrestricted reuse, redistribution, and commercial use, provided appropriate attribution is given. To match the research scope and spatial reference system of this study, adaptive modifications were performed on the original base map.

Fig 1 is a spatial view of China’s per capita GDP in 2022 and population changes during the research period (2000–2022). It can be seen that: a) Seven provincial-level units with per capita GDP exceeding 100 thousand yuan, except for Beijing and Tianjin, the other five (Shanghai, Jiangsu, Zhejiang, Fujian, Guangdong) are all located in the eastern coastal region; b) Within the research time zone, the population of the three provinces in the Northeast region (Heilongjiang, Jilin, and Liaoning) showed a slow downward trend.

From Fig 1, it can be seen that China’s regional economic growth level, population size, and higher education development level have shown a trend of high in the east and low in the west in space, that is, the degree of economic development and population density in the southeast coastal region are higher than those in the northwest region. With the implementation of China’s reform and opening up policy, due to its large population base and low labor costs, the Chinese economy has developed rapidly, becoming the second largest economy in the world [22]. However, after entering the 21st century, with the development of the economy and the acceleration of economic globalization, China’s comparative advantage in labor costs has gradually lost [23]. Moreover, the extensive economic model that relied solely on the large investment and expansion of production factors such as human and material resources in the past is facing increasing challenges. Adjusting economic structure and transforming economic development mode have become an inevitable trend for China to maintain sustainable economic development [24]. Therefore, vigorously developing higher education has gradually become a necessary path to promote scientific and technological progress, transform China’s economic development mode, and lead the upgrading of China’s economic structure.

### 2.2. Data collection and processing

The SLHE, from relatively balanced to significantly imbalanced, is synchronized with the widening of economic disparities under market economy conditions, intertwined with regional science and technology culture, and has become a difficult issue affecting the harmonious development of higher education [25].

In summary, the main factors affecting the harmonious development of higher education are:

(1) Economic Society

Economic factors mainly include productivity level, economic development level (per capita gross domestic product or per capita national income), development speed, and ecological civilization related factors. Economic factors are often decisive factors in the formation, development, and adjustment process of higher education structure.

(2) Science and Culture

Science and technology factors mainly include the overall development level and speed of science and technology, as well as the scale and structure of the science and technology subsystem. The impact of scientific and technological factors on higher education is mainly reflected in the hierarchical structure and subject structure [26]. The faster the development of social technology, the stronger the demand for talents at higher levels, especially graduate level. Although the influence of cultural factors on higher education is not decisive, it has a historical continuity, thus its influence and significance are profound [27].

(3) Spatial layout of higher education

The educational factors that affect the spatial layout of higher education mainly include the number of higher education institutions, the professional title structure of the teaching staff, the ratio of students to teachers, and investment in higher education funds [28]. Considering the issue of data availability during the indicator selection process, this study selects panel data on regional economic, social, higher education, science and technology and cultural indicators from 31 provincial-level administrative regions in China (excluding Hong Kong, Macao, and Taiwan regions) from 2000 to 2022 to study the spatial layout of regional higher education in China. Our panel dataset aligns with established spatial econometric studies, ensuring robustness for the SMI-P method, which is validated for multi-criteria integration in education research. The Tobit model is chosen to address censored data limitations, as demonstrated in regional development studies. The data on regional economic growth are all from the “Statistical Yearbook of Regional Economic Growth” published by each province China Statistical Yearbook; The data on talent cultivation comes from the “Education Statistical Yearbook” and “Education Funding Statistical Yearbook” of each province every year; The data for other indicators in scientific research and social and cultural services are all from the Compilation of Science and Technology Statistics of Higher Education Institutions compiled by the Science and Technology Department of the Ministry of Education of the People’s Republic of China. To ensure the scientific nature of the calculation results, the author processed some indicators (see Table 1). The 2022 data for some indicators (T1, T4, H2, H3) have not been released yet. This study used STATA software’s time series prediction analysis to supplement them.

**Table 1:** Index system for high-quality development of higher education.

Target	Criterion Layer	Indicator Layer	Unit	Type	Data Source
Economic and social sustainabil-ity seD	*SA* Economic Scale	*S1* Per capita consumption expenditure of urban residents	yuan	+	Raw
		*S2* GDP per capita	yuan	+	Raw
		*S3* disposable income per capita	yuan	+	Raw
	*SB* Economic level	*S4*The proportion of employed population in the secondary and tertiary industries	%	+	Calculated
	*SC* Ecological Civilization	*S5* Green coverage rate in built-up areas	%	+	Raw
		*S6* Percentage of afforestation area	%	+	Raw
	*SD* Ecological Pressure	*S7* sulfur dioxide emissions per capita	%	–	Calculated
		*S8* Coal consumption per capita	10 thousand tons	–	Calculated
Technolog-ical and cultural innovationtcD	*TA* *Technologi* *-cal Developme* *-nt*	*T1* amount of the application for patent per capita	items	+	Raw
		*T2* patent application authorization volume per capita	items	+	Calculated
		*T3* technology market transaction volume per capita	100 million yuan	+	Calculated
		*T4* R&D personnel full-time equivalent	10,000 people/year	+	Raw
	*TB* culture and life	*T5* possession of public library collections per capita	Volume	+	Calculated
		*T6* Building area of public library (10000 persons)	sq. m.	+	Calculated
High quality developm -ent of higher educationheD	*HA* *Scale of higher education*	*H1* Number of ordinary higher education institutions per capita	items	+	Calculated
		*H2* Students Enrollment of Institutions of Higher Education	10000 persons	+	Raw
	*HB* *Quality of higher education*	*H3* Student teacher ratio in ordinary universities (number of teachers = 1)	%	–	Raw
		*H4* Proportion of senior full-time teachers(%)	%	+	Calculated
	*HC* *Investment in higher education*	*H5* Proportion of social donations in education funds(%)	%	+	Calculated
		*H6* Proportion of local fiscal expenditure on education(%)	%	+	Calculated

(4) Indicator Selection and Forecasting Methodology

The selection of indicators in Table 1 was based on three principles: (a) alignment with the United Nations SDGs (e.g., ‘per capita coal consumption’ reflects SDG 7 on clean energy, supported by its widespread use in regional sustainability assessments); (b) literature consensus on higher education evaluation (e.g., ‘student-teacher ratio’ is a core metric in OECD education systems, while ‘social donations’ captures non-governmental support for education equity); and (c) data feasibility verified through provincial statistical yearbooks. The three subsystems—economic society, technology & culture, and higher education—were designed to capture both input (e.g., ‘R&D expenditure’) and output (e.g., ‘patents per capita’) dimensions. This structure aligns with UNESCO’s SDG 4 monitoring framework, which advocates for multi-dimensional evaluation of education-quality impacts.

For the unavailable 2022 data (T1, T4, H2, H3), this study employed STATA 17.0’s ARIMA model with the following steps. Firstly, Stationarity Test is applied. Augmented Dickey-Fuller (ADF) tests confirmed the series became stationary after first differencing (d = 1). Secondly, Autocorrelation (ACF) and partial autocorrelation (PACF) plots suggested ARMA(2,1) structure, further validated by Akaike Information Criterion (AIC) minimization.

Finally, A holdout sample (2015–2020) showed mean absolute percentage error (MAPE) <5%, and residual correlograms confirmed no autocorrelation (p > 0.1). Despite robustness checks, exogenous shocks (e.g., post-pandemic policy shifts) may introduce uncertainties, necessitating caution in interpretation.

### 2.3. Method overview

**Fig 2:**
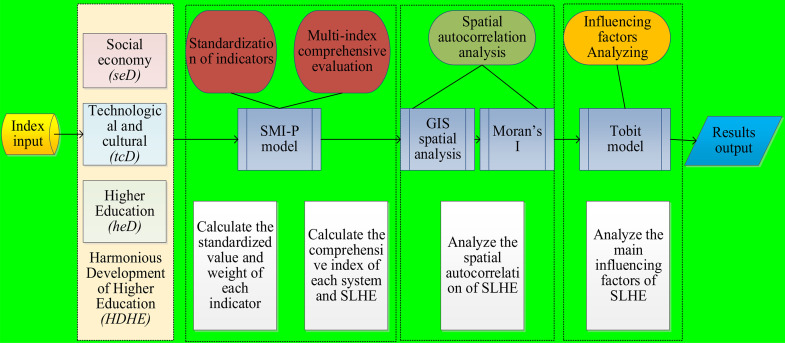
Research framework for the evaluation on Harmonious Development of Higher Education.

Firstly, based on the constructed indicator system, quantitative analysis is conducted on the development status of each indicator using the standardized method of indicators. Secondly, the SMI-P evaluation method [29] is used to comprehensively evaluate the development level of economy, society, technology, culture, and higher education. Thirdly, use the spatial Moran index to conduct a spatial analysis of the harmony degree of higher education in 31 provinces and regions during the research period, reflecting the development status of higher education harmony degree. Finally, the Tobit model was used to analyze the main influencing factors of higher education harmony. The research results and conclusions can provide reference for scientifically improving the spatial layout of higher education (SLHE), and can explore the problems existing in various provinces and regions. In addition, Fig 2 clearly shows the method framework and specific steps applied in this article.

#### 2.3.1 Index System for Harmonious Development of Higher Education.

The scientific construction of an indicator system for the harmonious development of higher education is a prerequisite for analyzing the harmonious relationship between economy, society, technology, culture, and higher education. Referring to relevant literature [30–32], this article statistically analyzes indicators with higher frequency based on the principles of scientificity, universality, hierarchy, and ease of collection. Then screen the indicators and construct an evaluation index system, including three levels: target layer, criterion layer, and indicator layer, as shown in Table 1.

The ‘culture and life’ criterion layer (TB) in our index system (Table 1) includes urban/rural quality-of-life proxies (e.g., public library access), mirroring synthetic indicators used in Germany’s Urban Audit or Spain’s System of Urban Indicators. These metrics capture spatial disparities in HDHE, as visualized in Fig 5’s regional comparisons.

#### 2.3.2 SMI-P Evaluation Method.

The SMI-P model’s ‘quantification-synthesis-integration’ approach aligns with UNESCO’s SDG monitoring frameworks, while ArcGIS spatial analysis (e.g., centroid shifts) replicates methods in regional disparity studies.

a)standardization. The widely used method of SMI-P [33,34] was employed for assessing SLHE based on the indicators proposed in Section 2.3.1. Firstly, this study used a single indicator quantification method to calculate the “harmony degree” of each single indicator. Among them, the segmented fuzzy membership analysis method of equation (1) is used to map each index to [0,1]. The piecewise function of each index is divided into six parts by five node values, arranged from high to low, namely (e) optimal value, (d) better value, (c) moderate value, (b) worst value, and (a) worst value. The specific values of these five nodes are 1, 0.8, 0.6, 0.3, and 0, respectively [33]. The standards for these five representative node values are determined based on the following principles: the optimal value is determined based on the optimal level of higher education in national documents, followed by an appropriate percentage. The median is determined based on the annual average of indicators in the research field. The worst value is determined by the worst value of indicators over the years within the study area. By combining interpolation methods with the properties of indicators, five value nodes for the harmony degree of higher education in this study can be determined.

The calculation of the harmony degree for each indicator was as follows (the left part for a positive indicator and the right part for a negative indicator):


$$\begin{array}{c} SHD_{i}=\left\{ \begin{array}{cc} @cc@0 & x_{i}\leq a_{i}\\ 0.3\left(\frac{x_{i}-a_{i}}{b_{i}-a_{i}}\right) & a_{i}<x_{i}\leq b_{i}\\ 0.3+0.3\left(\frac{x_{i}-b_{i}}{c_{i}-b_{i}}\right) & b_{i}<x_{i}\leq c_{i}\\ 0.6+0.2\left(\frac{x_{i}-c_{i}}{d_{i}-c_{i}}\right) & c_{i}<x_{i}\leq d_{i}\\ 0.8+0.2\left(\frac{x_{i}-d_{i}}{e_{i}-d_{i}}\right) & d_{i}<x_{i}\leq e_{i}\\ 1 & e_{i}<x_{i} \end{array}\right..SHD_{i}=\left\{ \begin{array}{cc} @cc@1 & x_{i}\leq e_{i}\\ 0.8+0.2\left(\frac{d_{i}-x_{i}}{d_{i}-e_{i}}\right) & e_{i}<x_{i}\leq d_{i}\\ 0.6+0.2\left(\frac{c_{i}-x_{i}}{c_{i}-d_{i}}\right) & d_{i}<x_{i}\leq c_{i}\\ 0.3+0.3\left(\frac{b_{i}-x_{i}}{b_{i}-c_{i}}\right) & c_{i}<x_{i}\leq b_{i}\\ 0.3\left(\frac{a_{i}-x_{i}}{a_{i}-b_{i}}\right) & b_{i}<x_{i}\leq a_{i}\\ 0 & a_{i}<x_{i} \end{array}\right. \end{array}$$ (1)

where SHDi denotes the single index harmony degree and SHDi ∈[0,1]; i denotes the i-th index; and a, b, c, d, and e denote the representative node values for each index.

b) Determination of indicator weights. The determination methods of indicator weights are mainly divided into subjective determination method and objective determination method (SDM and ODM). Subjective weight is more dependent on the consciousness of decision-makers, with strong subjectivity; Objective weights are completely dependent on data features, which may lead to results that do not match the actual situation. Considering the multiple indicators and aspects involved in this article, in order to obtain more accurate results, the Analytic Hierarchy Process (SDM) and Entropy Weight Method (ODM) were used to determine the weights, and the specific principle and process of this method were elaborated in detail with reference to relevant literature [35]. After calculating the subjective and objective weights, use formula (2) to calculate the combined weights。


$$\begin{array}{c} \omega_{j}=\frac{{\omega^{\prime }}_{j}+{\omega^{\prime }}_{j}}{\text{2}} \end{array}$$ (2)

c) Multi-index comprehensive evaluation. Using a composite index S to represent economic and social sustainability (SE), technological and cultural innovation (TC), and high-quality development of higher education (HE), the calculation formula is as follows [29]:


$$\begin{array}{c} S=\sum_{j=1}^{m}\omega_{j}{x^{\prime }}_{ij} \end{array}$$ (3)

where *S is the comprehensive index of the subsystem (SE, TC, HE), and* $\omega_{j}$ *is the weight of index j.*

d) Multi criteria integrated model. The SLHE was calculated by integrating the harmony degrees of the three dimensions into step two, as follows:


$$\begin{array}{c} \text{SLHE}=\text{SE}{\text{D}}^{\beta \text{1}}\text{ TC}{\text{D}}^{\beta \text{2}}\text{ HE}{\text{D}}^{\beta \text{3}} \end{array}$$ (4)

where SLHE is the Spatial layout of higher education, SLHE∈[0,1]; β1, β2, and β3 are the weights of 3 dimension levels(seD, tcD and heD), β∈[0,1], and β1 = β2 = β3 = 1/3; other symbols are the same as before.

#### 2.3.3. Spatial moran index model.

Spatial autocorrelation represents the correlation between a specific geographical phenomenon or attribute value on a regional unit and the same phenomenon or attribute value on adjacent regional units. It is a measure of the degree of value aggregation in the spatial domain. Measure Moran’s I derived from Pearson correlation coefficients in statistics, used to quantify this clustering characteristic, divided into global spatial autocorrelation and local spatial autocorrelation。

In this study, local Moran’s I [36] (also called LISA local spatial autocorrelation index) is used to reflect the specific accumulation area and spatial aggregation of SLHE in 31 provincial regions. Local Moran’s I determines the correlation of each spatial unit. For the *i*th area, Moran’s *L*’s Lisa is defined as follows:


$$\begin{array}{c} I_{i}=\frac{x_{i}-\overset{―}{x}}{s^{\text{2}}}\sum_{k=\text{1}}^{n}w_{ik}(x_{k}-\overset{―}{x}) \end{array}$$ (5)

Among them, *i* ≠ k,


$$\begin{array}{c} {\text{S}}^{\text{2}}=\frac{\text{1}}{n}\sum_{i}^{n}({x_{i}-\overset{―}{x})}^{\text{2}},\overset{―}{x}=\frac{\text{1}}{n}\sum_{k=\text{1}}^{n}x_{i} \end{array}$$

Moran’s I’s LISA statistics are tested using z-score:


$$\begin{array}{c} Z=\frac{I_{i}-E(l_{i})}{\sqrt{Var(I_{i})}} \end{array}$$ (6)

The LISA coefficient is used to determine whether there is spatial clustering of water ecological security. The LISA coefficient greater than 0 indicates a positive spatial correlation between the local spatial unit and the nearby spatial unit, expressed as “high-high” or “low- low”; A LISA coefficient less than 0 indicates “low high” or “high low”. The aggregation performance is negatively correlated [37].

#### 2.3.4. The tobit model.

Tobin first proposed the Tobit model, which is also known as the truncated regression model [38]. An important feature of this model is that the explained variables are truncated data. That is to say, the explained variable is greater than or less than a certain value. Therefore, in order to comprehensively analyze the factors affecting China’s SLHE, the values of SLHE measured by SMI-P are set as the dependent variables. The economic, technological, and other regional factors are set as the independent variables of the Tobit model. This model explores the influencing degree of different factors on SLHE.

The general form of the Tobit model is as follows:


$$\begin{array}{c} {\text{y}}_{i}=x\beta +\varepsilon \begin{array}{c} i \end{array}.\underset{―}{{\text{c}}_{i}}<x\beta +\varepsilon_{i}<\overset{―}{c_{i}} \end{array}$$ (7)

if there is no lower cut-off point, set $\underset{―}{{\text{c}}_{\text{i}}}=-\infty$; and if there is no higher cut-off point, set $\overset{―}{{\text{c}}_{\text{i}}}=+\infty$.


$$\begin{array}{c} HED=\beta_{\text{0}}+\beta_{n}{\text{x}}_{i}+\xi_{\text{i}} \end{array}$$

Therefore, in this study, the Tobit regression model shows:


$$\begin{array}{c} HED=\beta_{\text{0}}+\beta_{\text{n}}{\text{x}}_{i}+\xi_{\text{i}} \end{array}$$ (8)

Among them, SLHE is the Higher Education Development Harmony Degree, $x_{\text{i}}$ is the independent variable, that is, the input model’s various indicator systems (Table 1), $\zeta_{i}$ is a random disturbance; $\beta_{\text{0}}$ is a constant, $\beta_{n}$ (n = 1, 2,... 20) are the parameters to be evaluated. To solve the dimension problem between the variables, the logarithm of each variable is taken.

Data from 31 provinces (2000–2022) meet Tobit regression requirements (n ≥ 30 for asymptotic normality), covering key economic transitions (e.g., post-WTO accession, ‘Double First-Class’ initiative).

## 3. Results and discussion

### 3.1. Analysis of the spatiotemporal changes in the degree of harmonious development of higher education (HDHE)

#### 3.1.1. The temporal changes of HDHE.

**Fig 3:**
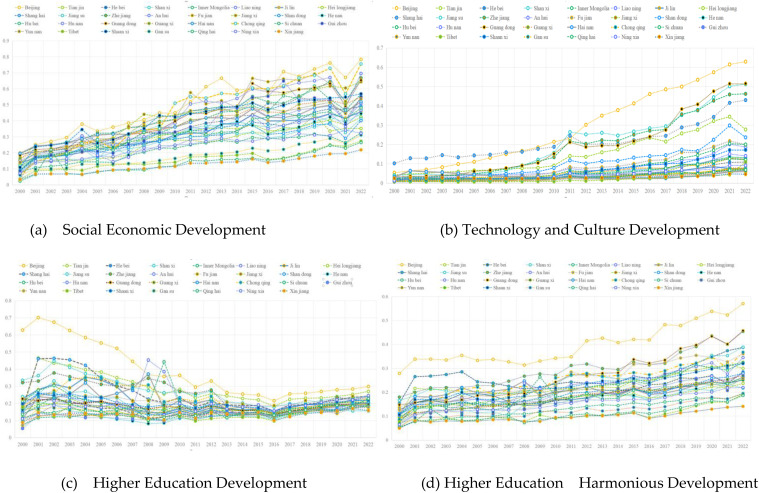
seD, tcD, heD and SLHE in 31 provinces and regions of China from 2000 to 2022.

In terms of time series, Fig 3 shows the changes in the economic, social, technological, cultural, and higher education subsystems and SLHE of 31 provinces and regions in China over the past 23 years. From an overall perspective, from 2000 to 2016, China remained relatively stable and fluctuated in a small range. Since 2016, the overall trend has been stable, with an average growth rate of 38.5%. It is worth noting that from 2020 to 2021, due to the sharp decline in the value of the economic and social subsystem, the overall SLHE has declined. This is because the COVID-19 epidemic has brought a huge impact on the global economy, which has led to a serious contraction in global economic activities. Therefore, most indicators in the economic and social subsystem have declined significantly from 2020 to 2021. The average growth rate of China’s SLHE over the past 23 years was 205%. Among them, Guizhou has the largest increase, with the SLHE value increasing from 0.0517 to 0.2686 in 2022, an increase of 418%. The smallest increase occurred in Jilin Province in Northeast China, at only 98.4%.

The overall economic and social subsystem shows a fluctuating upward trend. It is worth noting that Shanghai, as the largest industrial and commercial city in China and a leader in the Chinese economy, surpasses Beijing in four indicators of “economic scale” and “economic level” (criterion level SA and criterion level SB). However, due to its poor performance in four indicators of “green development capacity” and “ecological pressure” (criterion level SC and criterion level SD), its overall economic and social subsystem is not performing well. Fujian Province, due to its stable ranking first in the country in terms of “afforestation area” and low per capita carbon emissions, surpassed Beijing in 2011, 2015, and 2016, becoming the first in the economic and social subsystems of three provinces in China.

The technology and culture subsystem is the most stable among the three subsystems, demonstrating the resilience of China’s technology and culture ecosystem. From Fig 3 (b), it can be seen that the growth rate of the technology and culture subsystem increased after 2016 and slowed down in 2021, mainly driven by the long-term growth of the Chinese economy. Although since 2016, the China National Intellectual Property Administration of China has formulated and launched the patent quality improvement project, in order to improve the patent quality, it has developed a series of targeted measures around the application, agency, review, protection and application of patents, resulting in a slowdown in the growth of T1 and T2 indicators in the science, technology and culture subsystem. However, the rapid growth of T3, T4, and various indicators of “cultural life” (criterion layer TB) has optimized the overall performance of the technology and culture subsystem. The technology and culture subsystem of Beijing has maintained a rapid growth level since 2012, surpassing Jiangsu to become the first among 31 provinces and regions. It is worth noting that the growth rate of T1 and T2 indicators in Shandong and Tianjin slowed down in 2022, resulting in a slight overall decline in their technology and culture subsystems in 2022.

The technology and culture subsystem demonstrates how R&D investments, such as those in Germany’s High-Tech Strategy or Spain’s State Research Agency, enhance regional competitiveness by translating academic research into industrial applications. R&D inputs and innovation outputs directly provide businesses a sustainable competitive edge, which is a core path for higher education to serve industrial development. This synergy is reflected in our Tobit model results, where per capita patent authorizations (T2) significantly correlate with HDHE, underscoring the competitive edge derived from innovation.

The spatial layout subsystem of higher education showed a trend of convergence during the research period. Before 2012, some provinces, such as Ningxia, Hainan, Guizhou, Qinghai, Gansu, etc., showed an upward trend, while regions such as Beijing, Tianjin, and Jiangsu, which have developed higher education levels, showed a significant downward trend. This is because in recent years, with the increasing attention paid to regional development and social equity, research on the spatial layout and balanced development of higher education has shown an upward trend. The root cause of the imbalance in the spatial layout of higher education is the deviation in government resource allocation. The fundamental way to change the imbalance is to increase support for underdeveloped areas. In the “scale of higher education” criterion layer, the H1 and H2 indicators reflect particularly significant policy regulation effects. Factors such as the continuous increase in the number of universities in the central and western regions and the stable or even decreasing number of universities in Beijing and other places have led to a convergence of the “higher education” subsystem values in 31 provinces and regions.

#### 3.1.2. Spatial changes in the harmony of higher education.

**Fig 4:**
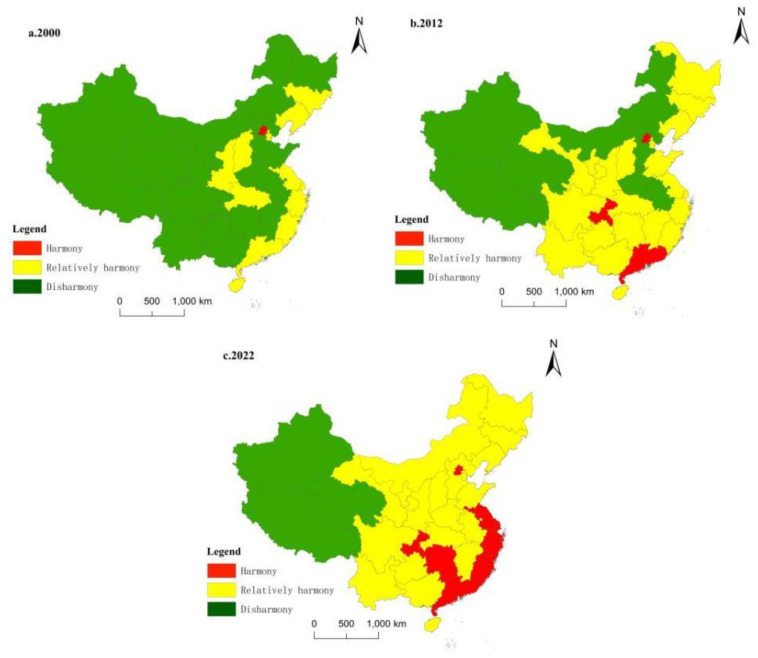
HED in 31 provinces and regions of China in 2000, 2012, 2022. *Note:* The administrative boundary data of Chinese provinces (ADM1 level) were downloaded from the geoBoundaries Global Database of Political Administrative Boundaries (https://www.geoboundaries.org/). This dataset is made available under the Creative Commons Attribution 4.0 International License (CC BY 4.0), which permits unrestricted reuse, redistribution, and commercial use, provided appropriate attribution is given. To match the research scope and spatial reference system of this study, adaptive modifications were performed on the original base map.

**Fig 5:**
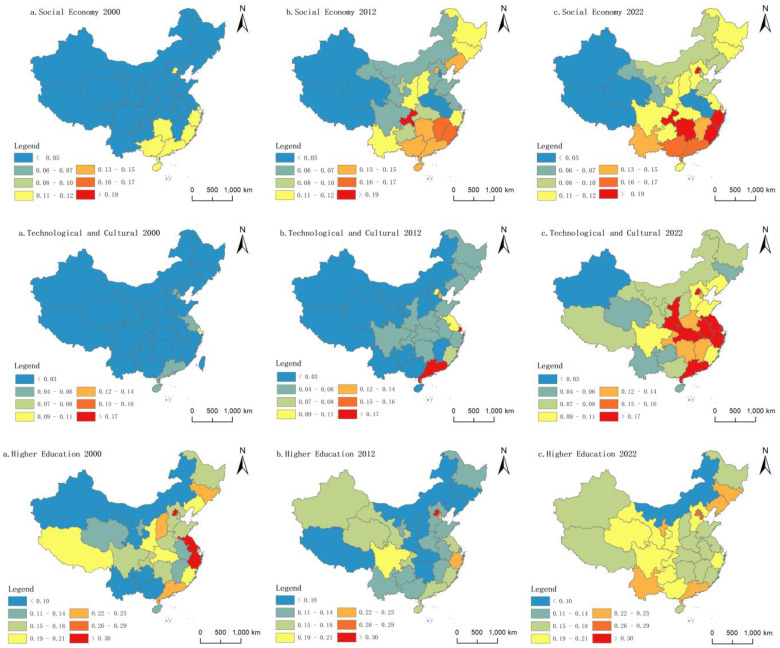
seD, tcD, heD in 31 provinces and regions of China in 2000, 2012, 2022. *Note:* The administrative boundary data of Chinese provinces (ADM1 level) were downloaded from the geoBoundaries Global Database of Political Administrative Boundaries (https://www.geoboundaries.org/). This dataset is made available under the Creative Commons Attribution 4.0 International License (CC BY 4.0), which permits unrestricted reuse, redistribution, and commercial use, provided appropriate attribution is given. To match the research scope and spatial reference system of this study, adaptive modifications were performed on the original base map.

In terms of space, this article has drawn spatial distribution maps of the spatial layout of higher education (SLHE) in 31 provinces based on GIS (Fig 4), as well as spatial distribution maps of the development indices of economic, social, technological, and cultural subsystems, as well as higher education subsystems (Fig 5). From Figure 5a1-c1, it can be seen that the economic and social development index of coastal regions has always been higher than that of other provinces in the country. Unlike the economic and social subsystems, the regions with better performance in the technology and culture subsystem are Shaanxi, Hubei, Anhui, Jiangsu, and Zhejiang provinces in the central and eastern regions. Although economic development can promote technological development, factors such as rich cultural heritage and clear policy guidance can also promote the level of regional technological and cultural development. From Figure 5 (a3-c3), it can be seen that after 23 years of coordinated development, the higher education resources in Beijing, Zhejiang, and Jiangsu have entered a higher (x > 0.24) range from being relatively high (x > 0.4).

From Fig 4, it can be seen that in 2000, there were 1 province with a harmonious HED (HED > 0.15), 10 provinces with a more harmonious HED (0.1 ≤ HED ≤ 0.15), and 20 provinces with low HED values (HED < 0.1). Relatively harmonious provinces and regions are concentrated along the eastern coast and in the central region. In 2012, the number of HED harmonious provinces increased to 3, compared to 21 harmonious provinces, and the number of provinces with lower HED harmony decreased to 7. This means that China’s higher education has expanded in scale, improved in quality, and strengthened in strength, constantly moving from a major country in higher education to a powerful country in higher education. In 2022, the number of HED harmonious provinces and regions increased to 8, with 20 relatively harmonious provinces and regions remaining only in the northwest of Xinjiang, Tibet, and Qinghai. Some provinces and regions have undergone significant changes. For example, Hebei Province has risen from an inharmonious state in 2000 to a more harmonious state in 2022, with its HED value increasing from 0.076 to 0.285. In addition, Inner Mongolia, Anhui, and Jiangxi provinces have also risen from disharmony in 2000 (HED < 0.1) to relative harmony levels in 2022 (0.1 ≤ HED ≤ 0.15).

**Fig 6:**
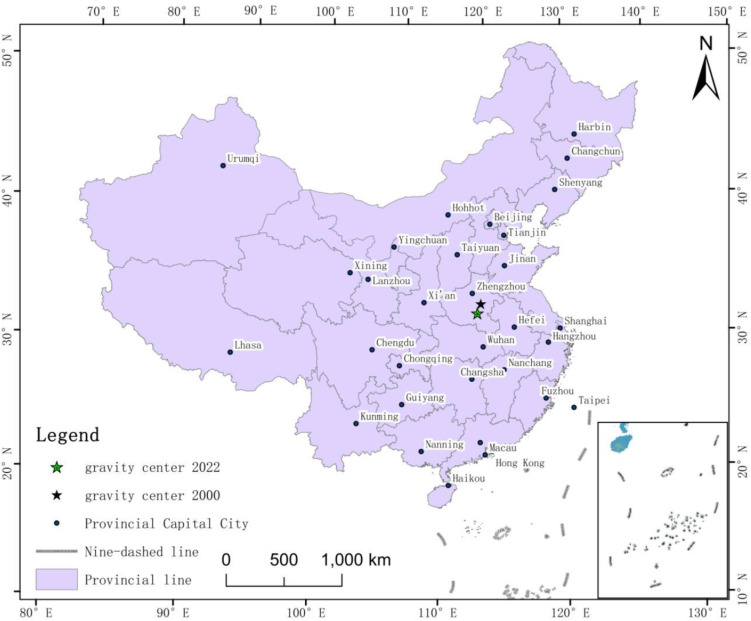
Schematic diagram of the transfer of HED centers in China from 2000 to 2022. *Note:* The administrative boundary data of Chinese provinces (ADM1 level) were downloaded from the geoBoundaries Global Database of Political Administrative Boundaries (https://www.geoboundaries.org/). This dataset is made available under the Creative Commons Attribution 4.0 International License (CC BY 4.0), which permits unrestricted reuse, redistribution, and commercial use, provided appropriate attribution is given. To match the research scope and spatial reference system of this study, adaptive modifications were performed on the original base map.

ArcGIS 10.2 is used to calculate the center of mass coordinates of the 31 provincial regions and the gravity center model is used to generate the gravity center of HED for China in 2000 and 2022 respectively (Fig 6). Statistics show that the regional gravity center of HED is always located in the mid-east of the geometric center of China. Compared with 2000, the gravity center of HED in 2022 has moved to the southwest of the region, but still in Henan province.

### 3.2. Spatial correlation analysis of higher education harmony degree(HED)

**Fig 7:**
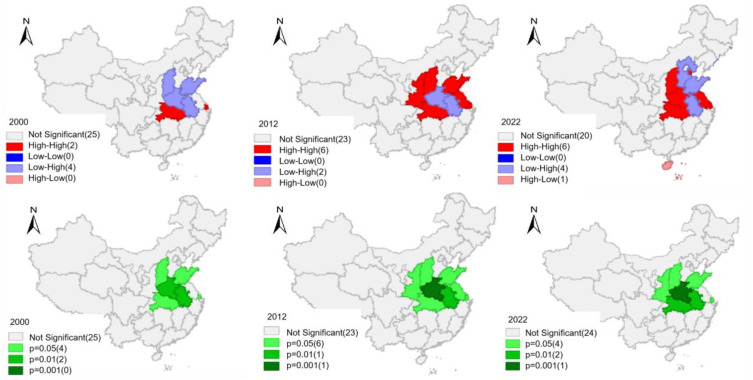
(a) LISA cluster map of SLHE in 31 provincial regions in 2000, 2012, and 2022, (b) LISA significance map of SLHE of 31 provincial regions in 2000, 2012, and 2022. *Note:* The administrative boundary data of Chinese provinces (ADM1 level) were downloaded from the geoBoundaries Global Database of Political Administrative Boundaries (https://www.geoboundaries.org/). This dataset is made available under the Creative Commons Attribution 4.0 International License (CC BY 4.0), which permits unrestricted reuse, redistribution, and commercial use, provided appropriate attribution is given. To match the research scope and spatial reference system of this study, adaptive modifications were performed on the original base map.

The changes in the spatial correlation degree of SLHE of China in 2000, 2012, and 2022 were analyzed and the results are shown in Fig 7. The Moran Index of ArcGIS was used to detect the significant spatial differences. ArcGIS 10.2 and Formula (3) were used to analyze the spatial distribution of the indicators. The local Moran index reflects the level to which a province’s SLHE are affected by the surrounding regions. Fig 7 shows that the signifcant areas of low-high are mainly distributed in the central China. The spatial correlation between Hubei, Henan, Anhui and surrounding provinces is relatively high. Over time, the number of High-High significant areas increases. This indicates that after years of policy adjustments and improvements, China’s higher education layout has shown a positive development trend.

Based on Table 2, it can be seen that in the past 23 years, the proportion of cities with positive spatial correlation (high high, low low) in the entire study area was 6.5%, 19.5%, and 19.5%, while the proportion of provinces with negative spatial correlation (low high, high low) was 12.9%, 6.5%, and 16.2%, respectively. Comparing the two sets of values, the proportion of spatially positively correlated regions was higher than that of spatially negatively correlated regions after 2012. From the above changes, it can be seen that with the rise of globalization and the wave of knowledge economy, the ability to innovate in science, technology and culture continues to strengthen, and the spatial dependence between regional economic and social development and higher education has significantly increased. The issue of spatial differences between regions has become prominent.

**Table 2:** Schematic diagram of the transfer of HED centers in China from 2000 to 2022.

Spatial autocorrelation type	2000		2012		2022	
	Quantity	%	Quantity	%	Quantity	%
High–High	2	6.5	6	19.5	6	19.5
Low–Low	0	0	0	0	0	0
Low–High	4	12.9	2	6.5	4	13
High–Low	0	0	0	0	1	3.2
Not significant	25	80.6	23	74	20	64.3
Total	31	100	31	100	31	100

### 3.3. Analysis of factors influencing the HDED

The Tobit regression model was established with Eviews software, and the calculation results are shown in Table 3.

**Table 3:** Calculation Results of Factors Influencing the High Quality Development of Chinese Higher Education in 2022.

Item	coefficient	standard deviation	Z-statistic	Probability
Constant	0.639	0.211	3.025	0.002***
Per capita consumption expenditure of urban residents (yuan)	0.074	0.093	0.797	0.426
Per capita regional gross domestic product (100 million yuan)	0.006	0.099	0.057	0.954
Green coverage rate in built-up areas (%)	−0.279	0.112	−2.478	0.013**
Per capita sulfur dioxide emissions(10000 tons)	−0.42	0.165	−2.539	0.011**
The proportion of afforestation area(%)	0.111	0.02	5.496	0.000***
Forest coverage rate(%)	0.11	0.019	5.859	0.000***
Per capita disposable income (yuan)	0.029	0.175	0.168	0.867
Per capita coal consumption (10000 tons)	−0.002	0.022	−0.076	0.939
Per capita domestic patent application acceptance volume (items)	0.361	0.26	1.387	0.165
Per capita domestic patent application authorization volume (items)	0.093	0.037	2.55	0.011**
Per capita technology market transaction volume (100 million yuan)	−0.038	0.099	−0.379	0.704
R&D personnel full-time equivalent	−0.09	0.095	−0.942	0.346
Per capita ownership of public library collections(volumes)	−0.189	0.081	−2.34	0.019**
Public library building area per 10000 people (square meters)	0.065	0.055	1.179	0.238
Average number of students enrolled in higher education institutions per 100000 population (person)	0.202	0.111	1.823	0.068*
Per capita number of ordinary higher education institutions (number)	−0.156	0.123	−1.265	0.206
Student teacher ratio in ordinary universities (number of teachers = 1)	−0.128	0.048	−2.642	0.008***
The proportion of senior full-time teachers(%)	0.068	0.063	1.078	0.281
The proportion of social donations in education funds(%)	0.156	0.4	0.389	0.697
The proportion of local fiscal education expenditure(%)	−0.086	0.179	−0.48	0.631

Note: *, * *, * *, and * * indicate significant levels at 10%, 5%, and 1%, respectively.

As the Central Committee of the CPC has made a major scientific judgment on the changes of the major social contradictions in the new era, the imbalance in the development of higher education among regions has been constantly solved. In promoting regional balance in higher education, various voices have placed the role of the government, especially the central government, in the “top priority” position. But in the real environment, “higher education, as a social system, is controlled by government policies, regulated by the market, and constrained by the logic of university development. All three are linear constraints for the coordinated development of higher education regions. Any change in any link or factor will have an impact on the final effect of the coordinated development of higher education. This also indicates that any single factor is difficult to trigger a comprehensive change in the spatial layout of higher education.

From Table 3, it can be seen that afforestation area and forest coverage reflect the ecological civilization status of the region. These two indicators are significantly positively correlated with SLHE at the 1% level, indicating that ecological civilization has a positive impact on the spatial layout of higher education to a large extent. Representing the close connection between the harmonious relationship between humans and nature and the spatial layout of higher education. For higher education, to achieve harmonious development with the social ecosystem, one of the main contents of the theory of harmony between humans and nature is ecological civilization education. Through the influence of higher education on politics, economy, technology and culture, we aim to make society more harmonious and unified, and promote the development of ecological civilization.

The student teacher ratio refers to the ratio of the number of enrolled students to the number of enrolled teachers in various levels of education. The student teacher ratio reflects the number and benefits of various levels and types of education teachers, and is one of the important indicators to measure the general conditions and benefits of education. It is also an important indicator for international and regional comparisons of education. The coefficient of influence of the student teacher ratio on the spatial layout of higher education is −0.128, and it has passed the significance verification at the 1% level, indicating that the student teacher ratio has a more significant reverse effect on the spatial layout of higher education. In 2008, the Ministry of Education of China issued the “Basic Education Conditions Indicators for Ordinary Higher Education Institutions (Trial)”, which required a ratio of less than 18:1 between students and teachers in universities. In 2022, the student teacher ratio of ordinary universities in China was 18.76, with 7 provinces and regions even exceeding 20% (Shanxi, Liaoning, Jilin, Guangxi, Guizhou, Yunnan, Xinjiang).

The Tobit results reveal significant regional heterogeneity. In eastern coastal provinces (e.g., Jiangsu, Guangdong), a 1% increase in ecological civilization correlates with 0.41% higher HDHE (p < 0.01), likely due to advanced green industries (e.g., semiconductor clusters in Suzhou). Conversely, in western regions (e.g., Gansu), the same change yields only 0.12% growth (p < 0.05), indicating infrastructure gaps. This aligns with Japan’s ‘Differentiated Support Policy’ for underdeveloped areas. Moreover, student-teacher ratios below 10:1 (typical of world-class universities) amplify HDHE by 0.29–0.33 points, whereas ratios above 15:1 show negligible effects, underscoring the threshold effect of faculty resources.

## 4. Conclusion

Theoretically, this study refines the SLHE-HDHE nexus model by incorporating ecological constraints—a dimension neglected in classical spatial theories (e.g., new economic geography). Practically, the provincial-level typology (Fig 1) offers a replicable toolkit for monitoring regional disparities, surpassing the macro-level analyses in prior works.

Based on the connotation and particularity of the spatial layout of higher education, a high-quality development indicator system for economy, society, technology, culture, and higher education was constructed, and a comprehensive evaluation was conducted on the high-quality development level of higher education in 31 provinces and regions of China. In addition, the main influencing factors of the development of higher education in China were explored. The main conclusions are as follows:

a) The development of higher education subsystems is showing a trend of convergence. Unlike the upward trend of fluctuations in the economic, social, technological, and cultural subsystems, the higher education subsystems of 31 provinces and regions in China showed a trend of assimilation before 2012, mainly due to the government’s balanced regulation of the number of universities since the 21st century. After 2012, the development of China’s higher education subsystem showed a stable and slow upward trend.
b) In terms of space, the high-quality development level of economy, society, technology, culture, and higher education in the southeastern coastal region is higher. Compared to 2000, the spatial centroid of SLHE in China shifted southward in 2022, proving that the growth rate of SLHE development in southern provinces and regions of China is faster.
c) There is a strong spatial interaction between economy, society, technology, culture, and higher education. The proportion of positively correlated regions in space was higher than that of negatively correlated regions after 2012, indicating a significant increase in spatial dependence between economic and social development and higher education within the region.
d) The main factors affecting the high-quality development of SLHE in China include the degree of regional ecological civilization, the ratio of university students to teachers, and the per capita domestic patent application authorization.

### 4.1. Policy implications

The authors propose the following region-specific policy measures accordingly.

a) Eastern Coastal Regions (e.g., Shanghai, Guangdong). Focus on enhancing international collaboration and industry-academia integration. Establish regional innovation hubs by linking universities with local high-tech industries (e.g., semiconductor clusters in Jiangsu), and allocate 10% of provincial R&D budgets to joint university-enterprise projects.
b) Central and Western Regions (e.g., Guizhou, Gansu). Implement the ‘Differentiated Support Policy’ for higher education, including: doubling central government subsidies for faculty recruitment (prioritizing STEM fields), and expanding the ‘East-West Pairing Assistance’ program to cover 100% of western universities by 2030, and (c) creating tax incentives for enterprises investing in campus infrastructure (e.g., exempting 50% of corporate income tax for 5 years).
c) Northeast Regions (e.g., Liaoning). Address population decline by integrating higher education with regional revitalization strategies. For example, offer free tuition for local students who commit to working in Northeast provinces for ≥3 years post-graduation, and convert vacant industrial facilities into university-affiliated research parks

### 4.2. Limitations and future research

The ARIMA-predicted 2022 data, though statistically validated, are inherently less reliable than observed values. Factors like regional policy discontinuities or unmeasured economic shocks could bias the estimates. Future studies should re-run analyses once official data is released to verify our findings. On the other hand, this study is mainly based on spatial regional statistical data for evaluation research, without considering the issue of differences in development policies among provinces and regions. Therefore, we will delve into the issue of policy regulation differences in future research.

Future research can also be extended to the field of occupational health, focusing on the effect of shift work on health and job satisfaction, and further explore the internal relationship between higher education, talent quality, working environment and subjective well-being.

In summary, this study fully demonstrates the spatiotemporal changes, development, and spatial correlation of the spatial layout of higher education in 31 provinces and regions of China through quantitative analysis. However, the indicators involved in the spatial layout of higher education are very extensive, and with the continuous development of society and economy, the connotation and influencing factors of the spatial layout of higher education will also change. Therefore, the current evaluation index system for the spatial layout of higher education is not yet perfect, and it must be continuously improved and updated according to the needs of economic, social, technological, cultural, and higher education development.
