# Supplementary material for: Research on the evaluation and spatial layout of high quality development of higher education in China
Source: PLoS One. 2026 Jul 9;21(7):e0347744. doi: 10.1371/journal.pone.0347744 (PMC13349139; doi:10.1371/journal.pone.0347744)
Supplement: Attachment — Submitted filename: a rebuttel letter.docx [file pone.0347744.s002.docx]

Dear Editor and Reviewers:

We appreciate your positive and constructive comments, suggestions and language corrections on our manuscript entitled “Research on the Evaluation and Spatial Layout of High Quality Development of Higher Education in China (PONE-D-25-16301)”.

**Journal Requirements:**

1. When submitting your revision, we need you to address these additional requirements.

Please ensure that your manuscript meets PLOS ONE's style requirements, including those for file naming. The PLOS ONE style templates can be found at

https://journals.plos.org/plosone/s/file?id=wjVg/PLOSOne_formatting_sample_main_body.pdf and

https://journals.plos.org/plosone/s/file?id=ba62/PLOSOne_formatting_sample_title_authors_affiliations.pdf

**Response:** Thank you. The manuscript has been reformatted to meet PLOS ONE's style requirements.

1.Main document is in DOC format with integrated tables (removed separate table files).

2.Supplementary materials (e.g., raw data) are uploaded as "Supporting Information" files, labeled as S1_Dataset.xlsx.

3.Line numbers and page numbers are continuous throughout the manuscript.

2. Please include your tables as part of your main manuscript and remove the individual files. Please note that supplementary tables (should remain/ be uploaded) as separate "supporting information" files.
3. Thank you for stating in your Funding Statement:

This work was supported by The 2021 Henan Higher Education Teaching Reform Research and Practice Project (2021SJGLX016) , the National Education and Science Planning Project (DIA230427), the 2024 Henan Province University Humanities and Social Sciences General Project (2024-ZZJH-412), the Open Project of Henan Provincial Key Laboratory of Water Conservancy Engineering Safety Technology (No. KJZX-JS-ZY- 202403), the High level talent research project launched by North China University of Water Resources and Electric Power (No. 202209007) and North China University of Water Resources and Electric Power New Era Water Control Social Science Research Institute "Open list" project (24B-01-02).

Please provide an amended statement that declares *all* the funding or sources of support (whether external or internal to your organization) received during this study, as detailed online in our guide for authors at http://journals.plos.org/plosone/s/submit-now.  Please also include the statement “There was no additional external funding received for this study.” in your updated Funding Statement.

Please include your amended Funding Statement within your cover letter. We will change the online submission form on your behalf.

4.Thank you for stating the following in the Acknowledgments Section of your manuscript:

This work was supported by The 2021 Henan Higher Education Teaching Reform Research and Practice Project (2021SJGLX016) , the National Education and Science Planning Project (DIA230427), the 2024 Henan Province University Humanities and Social Sciences General Project (2024-ZZJH-412), the Open Project of Henan Provincial Key Laboratory of Water Conservancy Engineering Safety Technology (No. KJZX-JS-ZY- 202403), the High level talent research project launched by North China University of Water Resources and Electric Power (No. 202209007) and North China University of Water Resources and Electric Power New Era Water Control Social Science Research Institute "Open list" project (24B-01-02).

We note that you have provided funding information that is not currently declared in your Funding Statement. However, funding information should not appear in the Acknowledgments section or other areas of your manuscript. We will only publish funding information present in the Funding Statement section of the online submission form.

Please remove any funding-related text from the manuscript and let us know how you would like to update your Funding Statement. Currently, your Funding Statement reads as follows:

This work was supported by The 2021 Henan Higher Education Teaching Reform Research and Practice Project (2021SJGLX016) , the National Education and Science Planning Project (DIA230427), the 2024 Henan Province University Humanities and Social Sciences General Project (2024-ZZJH-412), the Open Project of Henan Provincial Key Laboratory of Water Conservancy Engineering Safety Technology (No. KJZX-JS-ZY- 202403), the High level talent research project launched by North China University of Water Resources and Electric Power (No. 202209007) and North China University of Water Resources and Electric Power New Era Water Control Social Science Research Institute "Open list" project (24B-01-02).

Please include your amended statements within your cover letter; we will change the online submission form on your behalf.

**Response:** The complete funding information is provided below and has been removed from the manuscript’s Acknowledgments section and both been written in the cover letter and been uploaded as an independent file.

**Funding Statement:**

This work was supported by:

- National Education and Science Planning Project (DIA230427)

- Henan Higher Education Teaching Reform Project (2021SJGLX016)

- Henan Province University Humanities Project (2026-ZZJH-093)

- Henan Water Conservancy Research Project (GG202515)

- Open Project of Henan Provincial Key Lab (No.KJZX-JS-ZY-202403)

- North China University of Water Resources High-level Talent Project (No. 202209007)

- North China University New Era Water Control Social Science Project (24B-01-02).

Declaration: No additional external funding was received, and no conflicts of interest exist.

5. We note that Figures 1, 4, 5, 6 and 7 in your submission contain map images which may be copyrighted. All PLOS content is published under the Creative Commons Attribution License (CC BY 4.0), which means that the manuscript, images, and Supporting Information files will be freely available online, and any third party is permitted to access, download, copy, distribute, and use these materials in any way, even commercially, with proper attribution. For these reasons, we cannot publish previously copyrighted maps or satellite images created using proprietary data, such as Google software (Google Maps, Street View, and Earth). For more information, see our copyright guidelines: http://journals.plos.org/plosone/s/licenses-and-copyright.

We require you to either present written permission from the copyright holder to publish these figures specifically under the CC BY 4.0 license, or remove the figures from your submission:

 a. You may seek permission from the original copyright holder of Figures 1, 4, 5, 6 and 7 to publish the content specifically under the CC BY 4.0 license.

 We recommend that you contact the original copyright holder with the Content Permission Form (http://journals.plos.org/plosone/s/file?id=7c09/content-permission-form.pdf) and the following text:

“I request permission for the open-access journal PLOS ONE to publish XXX under the Creative Commons Attribution License (CCAL) CC BY 4.0 (http://creativecommons.org/licenses/by/4.0/). Please be aware that this license allows unrestricted use and distribution, even commercially, by third parties. Please reply and provide explicit written permission to publish XXX under a CC BY license and complete the attached form.”

 Please upload the completed Content Permission Form or other proof of granted permissions as an "Other" file with your submission.

 In the figure caption of the copyrighted figure, please include the following text: “Reprinted from [ref] under a CC BY license, with permission from [name of publisher], original copyright [original copyright year].”

 b. If you are unable to obtain permission from the original copyright holder to publish these figures under the CC BY 4.0 license or if the copyright holder’s requirements are incompatible with the CC BY 4.0 license, please either i) remove the figure or ii) supply a replacement figure that complies with the CC BY 4.0 license. Please check copyright information on all replacement figures and update the figure caption with source information. If applicable, please specify in the figure caption text when a figure is similar but not identical to the original image and is therefore for illustrative purposes only.

The following resources for replacing copyrighted map figures may be helpful:

 USGS National Map Viewer (public domain): http://viewer.nationalmap.gov/viewer/

The Gateway to Astronaut Photography of Earth (public domain): http://eol.jsc.nasa.gov/sseop/clickmap/

Maps at the CIA (public domain): https://www.cia.gov/library/publications/the-world-factbook/index.html and https://www.cia.gov/library/publications/cia-maps-publications/index.html

NASA Earth Observatory (public domain): http://earthobservatory.nasa.gov/

Landsat: http://landsat.visibleearth.nasa.gov/

USGS EROS (Earth Resources Observatory and Science (EROS) Center) (public domain): http://eros.usgs.gov/#

Natural Earth (public domain): http://www.naturalearthdata.com/

**Response:** We have also filled in the 'content-permission-form' required by the journal, please review.

6. Please provide a complete Data Availability Statement in the submission form, ensuring you include all necessary access information or a reason for why you are unable to make your data freely accessible. If your research concerns only data provided within your submission, please write "All data are in the manuscript and/or supporting information files" as your Data Availability Statement.

**Response:** The following statement has been uploaded as an independent file named “Data Availability Statement”.

Additional Editor Comments:

We have now received all three reviewer reports, which provide different perspectives on your work. Overall, I believe this study is valuable. Please revise it carefully according to the opinions of the three reviewers, and we will conduct another evaluation of the manuscript.
