# Supplementary material for: Research on the evaluation and spatial layout of high quality development of higher education in China
Source: PLoS One. 2026 Jul 9;21(7):e0347744. doi: 10.1371/journal.pone.0347744 (PMC13349139; doi:10.1371/journal.pone.0347744)
Supplement: Attachment — Submitted filename: Response to Reviewer Comments.doc [file pone.0347744.s005.doc]

Dear Editor and Reviewers:

We appreciate your positive and constructive comments, suggestions and language corrections on our manuscript entitled “Research on the Evaluation and Spatial Layout of High Quality Development of Higher Education in China (PONE-D-25-16301)”.

We have revised the manuscript carefully and thoroughly according to your advice. The main revisions are as follows:

We have provided a wider and more complete overview of the research field.

We have added and clarified the link among internationalization, learning organization, competitive edge, and research and development (R&D).

We have included discussions related to typical regions and countries such as the European Union, Spain, Germany, etc.

We have added the following topics as suggested:

How research and development provide businesses a competitive edge;

Efforts to innovate favouring the absorption of foreign direct investment so as to achieve beneficial outcomes from innovation;

Synthetic indicators of quality of subjective life in rural and urban areas;

Effect of shift work on health and job satisfaction.

All the revisions have been marked in the manuscript. We hope the revised version can meet the requirements of the journal. We sincerely thank you for your valuable time and professional comments.

Thank you again for your great efforts and continuous support. We look forward to your favorable reply.

Sincerely,

Wen Li

**full contact details:**

Correspondence to Wen Li:

Email: liwen@ncwu.edu.cn

**place of employment:** A teacher in North China University of Water Resources and Electric Power in China, Ph. D

**postal address:** North China University of Water Resources and Electric Power, No.136, Jinshui East Road, Zhengzhou 450046, P. R. China
