# Supplementary material for: Research on the evaluation and spatial layout of high quality development of higher education in China
Source: PLoS One. 2026 Jul 9;21(7):e0347744. doi: 10.1371/journal.pone.0347744 (PMC13349139; doi:10.1371/journal.pone.0347744)
Supplement: Attachment — Submitted filename: Response to Reviewer Comments.docx [file pone.0347744.s006.docx]

Dear Reviewer，

We are deeply grateful for your professional, rigorous, and constructive comments on our manuscript entitled “Research on the Evaluation and Spatial Layout of High Quality Development of Higher Education in China (PONE-D-25-16301)”. We have carefully revised the manuscript in full accordance with your suggestions, and all revisions are clearly marked with tracks in the revised version for your easy checking.

Our point‑to‑point responses are as follows:

***Comment 1***

Some improvements could be made. Authors should provide a wide and complete overview in journals indexed in Journal Citation Report.

***Response***

We have supplemented and strengthened the comprehensive and systematic literature overview to meet the academic standards and narrative requirements of JCR‑indexed journals.

We expanded the theoretical framework connecting higher education spatial layout, economic society, and technological culture.

We clarified the research gaps and academic contributions more explicitly.

We strengthened the logical chain and international perspective to enhance readability and academic completeness for international readers.

**Revision location:** Section 1 Introduction

***Comment 2***

They could include the link among internationalization, learning organization, competitive edge, research and development.

***Response***

We have clearly established the internal logical link among internationalization, learning organization, competitive edge, and R&D.We added a clear narrative:Internationalization promotes cross‑border academic cooperation; learning organization enhances continuous innovation capability; R&D in universities helps enterprises form core competitiveness; and competitiveness further drives the high‑quality development of higher education.

**Revision location:** Section 1 Introduction

***Comment 3***

So, they could mention the following related topics in geographical areas such as European Union, Spain, Germany, among others:

1) How research and development provide businesses a competitive edge.

***Response***

We added content explaining that university R&D input and innovation output directly enhance industrial technology level and help enterprises obtain sustainable competitive advantage, and compared it with typical innovation practices in the European Union, Spain, and Germany.

**Revision location:** Section 3.1.1 The Temporal Changes of HDHE

2) Efforts to innovate favouring the absorption of foreign direct investment so as to achieve beneficial outcomes from innovation.

***Response***

We supplemented the view that regional innovation capacity helps attract FDI, and FDI further promotes knowledge spillover and technology transfer to amplify innovation benefits, and noted that this model has been widely used in the European Union, Spain, Germany, and other regions.

**Revision location:** Section 1 Introduction

3) Synthetic indicators of quality of subjective life in rural and urban areas.

***Response***

We pointed out that the “culture and life” module in our index system includes synthetic indicators reflecting subjective life quality in urban and rural areas, which is consistent with the indicator systems used in the European Union, Spain, and Germany.

**Revision location:** Section 2.3.1 Index System for Harmonious Development of Higher Education

4) Effect of shift work on health and job satisfaction.

***Response***

We added this topic as an important direction for future research, suggesting that future research can be extended to occupational health, exploring the effect of shift work on health and job satisfaction, as well as its relationship with higher education, talent quality, and well‑being.

**Revision location:** Section 4.2 Limitations and Future Research

***Comment 4***

I wish the authors the best.

***Response***

Thank you sincerely for your kindness and valuable guidance! We have completed all revisions earnestly and hope the revised manuscript can meet the standards of the journal.

Best regards,

Corresponding Author: Wen Li

**full contact details:**

Correspondence to Wen Li:

Email: [liwen@ncwu.edu.cn](mailto:liwen@ncwu.edu.cn)

Date: 2026-03-25

**place of employment:** A teacher in North China University of Water Resources and Electric Power in China, Ph. D

**postal address:** North China University of Water Resources and Electric Power, No.136, Jinshui East Road, Zhengzhou 450046, P. R. China
