# Supplementary material for: Research on the evaluation and spatial layout of high quality development of higher education in China
Source: PLoS One. 2026 Jul 9;21(7):e0347744. doi: 10.1371/journal.pone.0347744 (PMC13349139; doi:10.1371/journal.pone.0347744)
Supplement: Peer review history

# Peer review: Decision Letter 0

**DOI:** 10.1371/journal.pone.0347744.r001

**Authors:** Ye Wei

4 Jun 2025

PONE-D-25-16301

Research on the Evaluation and Spatial Layout of High Quality Development of Higher Education in China

PLOS ONE

Dear Dr. Li,

Thank you for submitting your manuscript to PLOS ONE. After careful consideration, we feel that it has merit but does not fully meet PLOS ONE’s publication criteria as it currently stands. Therefore, we invite you to submit a revised version of the manuscript that addresses the points raised during the review process.

Please submit your revised manuscript by Jul 19 2025 11:59PM. If you will need more time than this to complete your revisions, please reply to this message or contact the journal office at plosone@plos.org. When you're ready to submit your revision, log on to https://www.editorialmanager.com/pone/ and select the 'Submissions Needing Revision' folder to locate your manuscript file.

Please include the following items when submitting your revised manuscript:

- A rebuttal letter that responds to each point raised by the academic editor and reviewer(s). You should upload this letter as a separate file labeled 'Response to Reviewers'.
- A marked-up copy of your manuscript that highlights changes made to the original version. You should upload this as a separate file labeled 'Revised Manuscript with Track Changes'.
- An unmarked version of your revised paper without tracked changes. You should upload this as a separate file labeled 'Manuscript'.

If you would like to make changes to your financial disclosure, please include your updated statement in your cover letter. Guidelines for resubmitting your figure files are available below the reviewer comments at the end of this letter.

If applicable, we recommend that you deposit your laboratory protocols in protocols.io to enhance the reproducibility of your results. Protocols.io assigns your protocol its own identifier (DOI) so that it can be cited independently in the future. For instructions see: https://journals.plos.org/plosone/s/submission-guidelines#loc-laboratory-protocols. Additionally, PLOS ONE offers an option for publishing peer-reviewed Lab Protocol articles, which describe protocols hosted on protocols.io. Read more information on sharing protocols at https://plos.org/protocols?utm_medium=editorial-email&utm_source=authorletters&utm_campaign=protocols.

We look forward to receiving your revised manuscript.

Kind regards,

Ye Wei, PhD

Academic Editor

PLOS ONE

**Journal Requirements:**

1. When submitting your revision, we need you to address these additional requirements.

Please ensure that your manuscript meets PLOS ONE's style requirements, including those for file naming. The PLOS ONE style templates can be found at

https://journals.plos.org/plosone/s/file?id=wjVg/PLOSOne_formatting_sample_main_body.pdf and

https://journals.plos.org/plosone/s/file?id=ba62/PLOSOne_formatting_sample_title_authors_affiliations.pdf

2. Please include your tables as part of your main manuscript and remove the individual files. Please note that supplementary tables (should remain/ be uploaded) as separate "supporting information" files.

3. Thank you for stating in your Funding Statement:

This work was supported by The 2021 Henan Higher Education Teaching Reform Research and Practice Project (2021SJGLX016) , the National Education and Science Planning Project (DIA230427), the 2024 Henan Province University Humanities and Social Sciences General Project (2024-ZZJH-412), the Open Project of Henan Provincial Key Laboratory of Water Conservancy Engineering Safety Technology (No. KJZX-JS-ZY- 202403), the High level talent research project launched by North China University of Water Resources and Electric Power (No. 202209007) and North China University of Water Resources and Electric Power New Era Water Control Social Science Research Institute "Open list" project (24B-01-02).

Please provide an amended statement that declares *all* the funding or sources of support (whether external or internal to your organization) received during this study, as detailed online in our guide for authors at http://journals.plos.org/plosone/s/submit-now.  Please also include the statement “There was no additional external funding received for this study.” in your updated Funding Statement.

Please include your amended Funding Statement within your cover letter. We will change the online submission form on your behalf.

4. Thank you for stating the following in the Acknowledgments Section of your manuscript:

This work was supported by The 2021 Henan Higher Education Teaching Reform Research and Practice Project (2021SJGLX016) , the National Education and Science Planning Project (DIA230427), the 2024 Henan Province University Humanities and Social Sciences General Project (2024-ZZJH-412), the Open Project of Henan Provincial Key Laboratory of Water Conservancy Engineering Safety Technology (No. KJZX-JS-ZY- 202403), the High level talent research project launched by North China University of Water Resources and Electric Power (No. 202209007) and North China University of Water Resources and Electric Power New Era Water Control Social Science Research Institute "Open list" project (24B-01-02).

We note that you have provided funding information that is not currently declared in your Funding Statement. However, funding information should not appear in the Acknowledgments section or other areas of your manuscript. We will only publish funding information present in the Funding Statement section of the online submission form.

Please remove any funding-related text from the manuscript and let us know how you would like to update your Funding Statement. Currently, your Funding Statement reads as follows:

This work was supported by The 2021 Henan Higher Education Teaching Reform Research and Practice Project (2021SJGLX016) , the National Education and Science Planning Project (DIA230427), the 2024 Henan Province University Humanities and Social Sciences General Project (2024-ZZJH-412), the Open Project of Henan Provincial Key Laboratory of Water Conservancy Engineering Safety Technology (No. KJZX-JS-ZY- 202403), the High level talent research project launched by North China University of Water Resources and Electric Power (No. 202209007) and North China University of Water Resources and Electric Power New Era Water Control Social Science Research Institute "Open list" project (24B-01-02).

Please include your amended statements within your cover letter; we will change the online submission form on your behalf.

5. We note that Figures 1, 4, 5, 6 and 7 in your submission contain map images which may be copyrighted. All PLOS content is published under the Creative Commons Attribution License (CC BY 4.0), which means that the manuscript, images, and Supporting Information files will be freely available online, and any third party is permitted to access, download, copy, distribute, and use these materials in any way, even commercially, with proper attribution. For these reasons, we cannot publish previously copyrighted maps or satellite images created using proprietary data, such as Google software (Google Maps, Street View, and Earth). For more information, see our copyright guidelines: http://journals.plos.org/plosone/s/licenses-and-copyright.

We require you to either present written permission from the copyright holder to publish these figures specifically under the CC BY 4.0 license, or remove the figures from your submission:

a. You may seek permission from the original copyright holder of Figures 1, 4, 5, 6 and 7 to publish the content specifically under the CC BY 4.0 license.

We recommend that you contact the original copyright holder with the Content Permission Form (http://journals.plos.org/plosone/s/file?id=7c09/content-permission-form.pdf) and the following text:

“I request permission for the open-access journal PLOS ONE to publish XXX under the Creative Commons Attribution License (CCAL) CC BY 4.0 (http://creativecommons.org/licenses/by/4.0/). Please be aware that this license allows unrestricted use and distribution, even commercially, by third parties. Please reply and provide explicit written permission to publish XXX under a CC BY license and complete the attached form.”

Please upload the completed Content Permission Form or other proof of granted permissions as an "Other" file with your submission.

In the figure caption of the copyrighted figure, please include the following text: “Reprinted from [ref] under a CC BY license, with permission from [name of publisher], original copyright [original copyright year].”

b. If you are unable to obtain permission from the original copyright holder to publish these figures under the CC BY 4.0 license or if the copyright holder’s requirements are incompatible with the CC BY 4.0 license, please either i) remove the figure or ii) supply a replacement figure that complies with the CC BY 4.0 license. Please check copyright information on all replacement figures and update the figure caption with source information. If applicable, please specify in the figure caption text when a figure is similar but not identical to the original image and is therefore for illustrative purposes only.

The following resources for replacing copyrighted map figures may be helpful:

USGS National Map Viewer (public domain): http://viewer.nationalmap.gov/viewer/

The Gateway to Astronaut Photography of Earth (public domain): http://eol.jsc.nasa.gov/sseop/clickmap/

Maps at the CIA (public domain): https://www.cia.gov/library/publications/the-world-factbook/index.html and https://www.cia.gov/library/publications/cia-maps-publications/index.html

NASA Earth Observatory (public domain): http://earthobservatory.nasa.gov/

Landsat: http://landsat.visibleearth.nasa.gov/

USGS EROS (Earth Resources Observatory and Science (EROS) Center) (public domain): http://eros.usgs.gov/#

Natural Earth (public domain): http://www.naturalearthdata.com/

6. Please provide a complete Data Availability Statement in the submission form, ensuring you include all necessary access information or a reason for why you are unable to make your data freely accessible. If your research concerns only data provided within your submission, please write "All data are in the manuscript and/or supporting information files" as your Data Availability Statement.

**Additional Editor Comments:**

We have now received all three reviewer reports, which provide different perspectives on your work. Overall, I believe this study is valuable. Please revise it carefully according to the opinions of the three reviewers, and we will conduct another evaluation of the manuscript.

[Note: HTML markup is below. Please do not edit.]

Reviewers' comments:

Reviewer's Responses to Questions

**Comments to the Author**

1. Is the manuscript technically sound, and do the data support the conclusions?

The manuscript must describe a technically sound piece of scientific research with data that supports the conclusions. Experiments must have been conducted rigorously, with appropriate controls, replication, and sample sizes. The conclusions must be drawn appropriately based on the data presented.

Reviewer #1: Partly

Reviewer #2: Partly

Reviewer #3: Partly

**********

2. Has the statistical analysis been performed appropriately and rigorously?

Reviewer #1: Yes

Reviewer #2: No

Reviewer #3: Yes

**********

3. Have the authors made all data underlying the findings in their manuscript fully available?

The PLOS Data policy requires authors to make all data underlying the findings described in their manuscript fully available without restriction, with rare exception (please refer to the Data Availability Statement in the manuscript PDF file). The data should be provided as part of the manuscript or its supporting information, or deposited to a public repository. For example, in addition to summary statistics, the data points behind means, medians and variance measures should be available. If there are restrictions on publicly sharing data—e.g. participant privacy or use of data from a third party—those must be specified.

Reviewer #1: No

Reviewer #2: Yes

Reviewer #3: Yes

**********

4. Is the manuscript presented in an intelligible fashion and written in standard English?

PLOS ONE does not copyedit accepted manuscripts, so the language in submitted articles must be clear, correct, and unambiguous. Any typographical or grammatical errors should be corrected at revision, so please note any specific errors here.

Reviewer #1: Yes

Reviewer #2: Yes

Reviewer #3: Yes

**********

5. Review Comments to the Author

Please use the space provided to explain your answers to the questions above. You may also include additional comments for the author, including concerns about dual publication, research ethics, or publication ethics. (Please upload your review as an attachment if it exceeds 20,000 characters)

**Reviewer #1:**  1.Timeliness and Completeness of Data:

The paper mentions that some 2022 data (e.g., T1, T4, H2, H3) were unavailable and were supplemented using STATA time-series forecasting. However, it does not justify the forecasting method or validate its accuracy. It is recommended to provide a detailed explanation of the forecasting methodology or explicitly note the limitations of the predicted data to avoid compromising the credibility of the conclusions.

2.Further Justification Needed for the Indicator System:

Although Table 1 presents a comprehensive set of indicators, the specific rationale for their selection (e.g., why "per capita coal consumption" was chosen over other pollution indicators) is not sufficiently explained. It is advisable to supplement the discussion with a literature review or expert consultation process to enhance the scientific validity of the indicators. Additionally, some indicators (e.g., "proportion of social donations in education funds") may face regional data availability challenges, and the paper should clarify how such issues were addressed.

3.Lack of Targeted Policy Recommendations:

While the conclusion highlights factors such as ecological civilization and student-teacher ratios, it does not propose differentiated policy suggestions based on regional disparities (e.g., tailored strategies for coastal vs. western regions). Providing region-specific policy insights would strengthen the study’s practical relevance.

4.Issues in English Writing and Citation Formatting:

Inconsistent terminology (e.g., mixing "higher education subsystem" and "HE subsystem").

Non-standard reference formatting (e.g., inconsistent author initials, journal names not italicized).

Grammatical issues in some sentences (e.g., unclear subject in the Abstract: "the degree of harmonious development among the three is determined").

A thorough language polish and strict adherence to PLOS ONE’s formatting guidelines are recommended.

5.The English names of cities in Figures 1, 3, and 6 need to be verified for accuracy, such as the spellings of Harbin, Taipei, and Hohhot.

**Reviewer #2:**  The manuscript presents an analysis of the spatial layout and development of higher education in China. While the topic is relevant, the manuscript fails to demonstrate significant innovation or marginal contribution to the existing literature on the topic of higher education development. MoreoverThe key concepts within the manuscript are confused and not clearly defined. For instance, the term "spatial layout of higher education SLHE" is used interchangeably with related but distinct concepts, leading to conceptual ambiguity. This lack of clarity undermines the rigor and coherence of the research. In addition, the selection of indicators used in the evaluation system is not sufficiently justified. The manuscript does not provide a clear rationale for why specific indicators were chosen over others, nor does it demonstrate that these indicators comprehensively capture the dimensions of higher education development being investigated. Additionally, the analysis of the results is overly simplistic and somewhat arbitrary. The interpretations drawn from the data lack depth and nuance, especially in chapter 3.3 Analysis of Factors Influencing the HDED. Given the aforementioned issues, I think the overall quality of the manuscript does not meet the publication standards of PLOS ONE.

**Reviewer #3:**  This paper tries to construct an indicator system for harmonious development of China’s higher education, and uses the method of “single indicator quantification-multi indicator synthesis-multi criteria integration” (SMI-P) to determine the harmonious development index of China’s higher education.

Authors should provide a wide and complete overview. They could include the link among learning organization, competitive edge, research and development. So, they could mention previous similar studies about How research and development provide businesses a competitive edge.

The hypotheses could appear as a result of literature review.

The data, model and methodology should be explained and justified based on the literature review. It should be justified whether the sample size is enough to apply the methodology and be able to interpret the results.

I wish the authors the best of success.

**********

6. PLOS authors have the option to publish the peer review history of their article (what does this mean?). If published, this will include your full peer review and any attached files.

If you choose “no”, your identity will remain anonymous but your review may still be made public.

**Do you want your identity to be public for this peer review?**  For information about this choice, including consent withdrawal, please see our Privacy Policy.

Reviewer #1: No

Reviewer #2: No

Reviewer #3: No

**********

[NOTE: If reviewer comments were submitted as an attachment file, they will be attached to this email and accessible via the submission site. Please log into your account, locate the manuscript record, and check for the action link "View Attachments". If this link does not appear, there are no attachment files.]

While revising your submission, please upload your figure files to the Preflight Analysis and Conversion Engine (PACE) digital diagnostic tool, https://pacev2.apexcovantage.com/. PACE helps ensure that figures meet PLOS requirements. To use PACE, you must first register as a user. Registration is free. Then, login and navigate to the UPLOAD tab, where you will find detailed instructions on how to use the tool. If you encounter any issues or have any questions when using PACE, please email PLOS at figures@plos.org. Please note that Supporting Information files do not need this step.

# Author comment: Author response to Decision Letter 1

**DOI:** 10.1371/journal.pone.0347744.r002

13 Aug 2025

Dear Editor and Reviewers:

We appreciate your positive and constructive comments, suggestions and language corrections on our manuscript entitled “Research on the Evaluation and Spatial Layout of High Quality Development of Higher Education in China (PONE-D-25-16301)”.

1. Response: Thank you. The manuscript has been reformatted to meet PLOS ONE's style requirements.

Main document is in DOC format with integrated tables (removed separate table files).

Supplementary materials (e.g., raw data) are uploaded as "Supporting Information" files, labeled as S1_Dataset.xlsx.

Line numbers and page numbers are continuous throughout the manuscript.

2.Response: The complete funding information is provided below and has been removed from the manuscript’s Acknowledgments section and both been written in the cover letter and been uploaded as an independent file.

Funding Statement:

This work was supported by:

- National Education and Science Planning Project (DIA230427)

- Henan Higher Education Teaching Reform Project (2021SJGLX016)

- Henan Province University Humanities Project (2026-ZZJH-093)

- Henan Water Conservancy Research Project (GG202515)

- Open Project of Henan Provincial Key Lab (No.KJZX-JS-ZY-202403)

- North China University of Water Resources High-level Talent Project (No. 202209007)

- North China University New Era Water Control Social Science Project (24B-01-02).

Declaration: No additional external funding was received, and no conflicts of interest exist.

3.Response: We have also filled in the 'content-permission-form' required by the journal, please review.

4.Response: The following statement has been uploaded as an independent file named “Data Availability Statement”.

Reviewer 1:

Response: Thank you for your positive and constructive review. We appreciate your time and efforts. The changes were highlighted with track changes for easy review.

We hope that the revision meets the requirement for publication.

1.Timeliness and Completeness of Data:

The paper mentions that some 2022 data (e.g., T1, T4, H2, H3) were unavailable and were supplemented using STATA time-series forecasting. However, it does not justify the forecasting method or validate its accuracy. It is recommended to provide a detailed explanation of the forecasting methodology or explicitly note the limitations of the predicted data to avoid compromising the credibility of the conclusions.

Response: Thank you for your suggestion. The paper mentions that some 2022 data (such as T1, T4, H2, H3) were supplemented through STATA time series prediction due to their non-public nature, but does not specify the specific steps of the prediction method or verify its accuracy.

The authors added a detailed explanation of the prediction method in the 2.2 Data Collection and Processing section, including model selection (ARIMA), parameter calibration (ADF test, lag order determination), and validation steps (MAPE error analysis), to enhance the transparency of the method.

The following explanation were added.

“(4) Indicator Selection and Forecasting Methodology

The selection of indicators in Table 1 was based on three principles: (a) alignment with the United Nations SDGs (e.g., ‘per capita coal consumption’ reflects SDG 7 on clean energy, supported by its widespread use in regional sustainability assessments); (b) literature consensus on higher education evaluation (e.g., ‘student-teacher ratio’ is a core metric in OECD education systems, while ‘social donations’ captures non-governmental support for education equity); and (c) data feasibility verified through provincial statistical yearbooks. The three subsystems—economic society, technology & culture, and higher education—were designed to capture both input (e.g., 'R&D expenditure') and output (e.g., 'patents per capita') dimensions. This structure aligns with UNESCO's SDG 4 monitoring framework, which advocates for multi-dimensional evaluation of education-quality impacts.

For the unavailable 2022 data (T1, T4, H2, H3), this study employed STATA 17.0's ARIMA model with the following steps. Firstly, Stationarity Test is applied. Augmented Dickey-Fuller (ADF) tests confirmed the series became stationary after first differencing (d=1) . Secondly, Autocorrelation (ACF) and partial autocorrelation (PACF) plots suggested ARMA(2,1) structure, further validated by Akaike Information Criterion (AIC) minimization.

Finally, A holdout sample (2015-2020) showed mean absolute percentage error (MAPE) <5%, and residual correlograms confirmed no autocorrelation (p>0.1). Despite robustness checks, exogenous shocks (e.g., post-pandemic policy shifts) may introduce uncertainties, necessitating caution in interpretation.”

(Page 13, Line 252)

In addition, it is necessary to do so in 4 Conclusion part clearly indicate potential errors in the predicted data. This supplement can both comply with academic standards and avoid conclusion disputes caused by data issues.

The following explanation were added.

“Theoretically, this study refines the SLHE-HDHE nexus model by incorporating ecological constraints—a dimension neglected in classical spatial theories (e.g., new economic geography). Practically, the provincial-level typology (Fig. 1) offers a replicable toolkit for monitoring regional disparities, surpassing the macro-level analyses in prior works.”

(Page 35, Line 590)

2.Further Justification Needed for the Indicator System:

Although Table 1 presents a comprehensive set of indicators, the specific rationale for their selection (e.g., why "per capita coal consumption" was chosen over other pollution indicators) is not sufficiently explained. It is advisable to supplement the discussion with a literature review or expert consultation process to enhance the scientific validity of the indicators. Additionally, some indicators (e.g., "proportion of social donations in education funds") may face regional data availability challenges, and the paper should clarify how such issues were addressed.

Response: Thank you for your suggestion. The selection of indicators in Table 1 was based on three principles: (a) alignment with the United Nations SDGs (e.g., ‘per capita coal consumption’ reflects SDG 7 on clean energy, supported by its widespread use in regional sustainability assessments); (b) literature consensus on higher education evaluation (e.g., ‘student-teacher ratio’ is a core metric in OECD education systems, while ‘social donations’ captures non-governmental support for education equity); and (c) data feasibility verified through provincial statistical yearbooks. (Page 13, Line 252)

3.Lack of Targeted Policy Recommendations:

While the conclusion highlights factors such as ecological civilization and student-teacher ratios, it does not propose differentiated policy suggestions based on regional disparities (e.g., tailored strategies for coastal vs. western regions). Providing region-specific policy insights would strengthen the study’s practical relevance.

Response: The authors added specific policy recommendations for the differentiated characteristics of different regions in China, such as the eastern coastal and western inland areas in the Conclusion section of the final manuscript.

“4.1 Policy Implications

The authors propose the following region-specific policy measures accordingly.

a)Eastern Coastal Regions (e.g., Shanghai, Guangdong). Focus on enhancing international collaboration and industry-academia integration. Establish regional innovation hubs by linking universities with local high-tech industries (e.g., semiconductor clusters in Jiangsu), and allocate 10% of provincial R&D budgets to joint university-enterprise projects.

b)Central and Western Regions (e.g., Guizhou, Gansu). Implement the ‘Differentiated Support Policy’ for higher education, including: doubling central government subsidies for faculty recruitment (prioritizing STEM fields), and expanding the ‘East-West Pairing Assistance’ program to cover 100% of western universities by 2030, and creating tax incentives for enterprises investing in campus infrastructure (e.g., exempting 50% of corporate income tax for 5 years).

c) Northeast Regions (e.g., Liaoning). Address population decline by integrating higher education with regional revitalization strategies. For example, offer free tuition for local students who commit to working in Northeast provinces for ≥3 years post-graduation, and convert vacant industrial facilities into university-affiliated research parks

4.2 Limitations and Future Research

The ARIMA-predicted 2022 data, though statistically validated, are inherently less reliable than observed values. Factors like regional policy discontinuities or unmeasured economic shocks could bias the estimates. Future studies should re-run analyses once official data is released to verify our findings. On the other hand, this study is mainly based on spatial regional statistical data for evaluation research, without considering the issue of differences in development policies among provinces and regions. Strategic learning as a dynamic capability that allows higher education institutions to break from current paths and renew their core competencies. This aligns with the paper's focus on subsystem interactions and spatial-temporal coordination. Knowledge management processes including strategic knowledge creation, dissemination, interpretation and implementation could be integrated into the indicator system, particularly in the technology & culture subsystem. Therefore, we will delve into the issue of policy regulation differences in future research.

In summary, this study fully demonstrates the spatiotemporal changes, development, and spatial correlation of the spatial layout of higher education in 31 provinces and regions of China through quantitative analysis. However, the indicators involved in the spatial layout of higher education are very extensive, and with the continuous development of society and economy, the connotation and influencing factors of the spatial layout of higher education will also change. Therefore, the current evaluation index system for the spatial layout of higher education is not yet perfect, and it must be continuously improved and updated according to the needs of economic, social, technological, cultural, and higher education development.” (Page 36, Line 618)

4.Issues in English Writing and Citation Formatting:

Inconsistent terminology (e.g., mixing "higher education subsystem" and "HE subsystem").

Non-standard reference formatting (e.g., inconsistent author initials, journal names not italicized).

Grammatical issues in some sentences (e.g., unclear subject in the Abstract: "the degree of harmonious development among the three is determined").

A thorough language polish and strict adherence to PLOS ONE’s formatting guidelines are recommended.

Response: We are sorry for our careless and thank you for your careful review. The following language and formatting issues had been addressed in the paper:

1. Inconsistent terminology. The “higher education subsystem” and “HE subsystem” have been unified into the full name “higher education subsystem”, but we remained the HE subsystem in tables for brief viewing and gave explanation for it;

2. Standardized Inconsistent author abbreviations and italicized the journal names;

3. Grammar issues. The subject of “the degree of harmonic development among the three is determined” in the abstract is ambiguous and has been changed to “this study determines the degree of harmonic development among the three subsystems” to clarify the subject;

4. Modify the format to meet the requirements of PLOS ONE.

5.The English names of cities in Figures 1, 3, and 6 need to be verified for accuracy, such as the spellings of Harbin, Taipei, and Hohhot.

Response: We are sorry for our careless and thank you for your careful review. The figures are verified accordingly and the names of cities are revised.

Reviewer 2:

The manuscript presents an analysis of the spatial layout and development of higher education in China.

Response: Thank you for your positive and constructive review. We appreciate your time and efforts, and acknowledgment was added in the revision. The changes were highlighted with track changes for easy review.

We hope that the revision meets the requirement for publication.

1.While the topic is relevant, the manuscript fails to demonstrate significant innovation or marginal contribution to the existing literature on the topic of higher education development. Moreover, The key concepts within the manuscript are confused and not clearly defined. For instance, the term "spatial layout of higher education SLHE" is used interchangeably with related but distinct concepts, leading to conceptual ambiguity. This lack of clarity undermines the rigor and coherence of the research.

Response: Thank you for the suggestion. The authors revised the manuscript accordingly.

�1�Rewrite innovative research at the end of the Introduction section

“While prior studies have extensively analyzed SLHE’s historical evolution [18] and policy optimization [19], this study contributes to the literature in three ways: (a) introducing a novel SMI-P-ArcGIS integrated framework to quantify HDHE’s spatial-temporal dynamics at provincial level, filling the gap in micro-scale empirical evidence; (b) identifying ‘ecological civilization’ as a key factor overlooked in existing SLHE models; and (c) proposing differentiated policy pathways for eastern-coastal vs. western-inland regions based on Tobit regression results, advancing the discourse on spatial justice in higher education.” ( Page 8. Line 145)

�2�Clearly define SLHE and differentiate related concepts at the beginning of Section 2.1

“The definition of SLHE refers to the distribution of higher education institutions' quantity, structure, and hierarchy across geographical/ administrative regions, and their dynamic interactions with regional socioeconomic development. This differs from 'balanced development' (equity-focused) or 'resource optimization' (efficiency-focused), as SLHE emphasizes spatial-temporal coordination among multiple subsystems (economic, technological, educational).

As for Conceptual Boundaries, which means SLHE is distinct from—but interdependent with—three related concepts: (i) HDHE measures outcome harmony among subsystems; (ii) regional equity evaluates access fairness; (iii) resource allocation focuses on input efficiency. Our study bridges these concepts by examining how SLHE shapes HDHE through spatial econometrics.”( Page 9. Line 177)

�3�Added content that strengthens the theoretical contribution in the Conclusion section

“Theoretically, this study refines the SLHE-HDHE nexus model by incorporating ecological constraints—a dimension neglected in classical spatial theories (e.g., new economic geography). Practically, the provincial-level typology (Fig. 1) offers a replicable toolkit for monitoring regional disparities, surpassing the macro-level analyses in prior works.”( Page 35. Line 589)

2.The selection of indicators used in the evaluation system is not sufficiently justified. The manuscript does not provide a clear rationale for why specific indicators were chosen over others, nor does it demonstrate that these indicators comprehensively capture the dimensions of higher education development being investigated.

Response: The following part has been added in 2.2 Data collection and processing:

“The selection of indicators in Table 1 was based on three principles: (a) alignment with the United Nations SDGs (e.g., ‘per capita coal consumption’ reflects SDG 7 on clean energy, supported by its widespread use in regional sustainability assessments); (b) literature consensus on higher education evaluation (e.g., ‘student-teacher ratio’ is a core metric in OECD education systems, while ‘social donations’ captures non-governmental support for education equity); and (c) data feasibility verified through provincial statistical yearbooks. The three subsystems—economic society, technology & culture, and higher education—were designed to capture both input (e.g., 'R&D expenditure') and output (e.g., 'patents per capita') dimensions. This structure aligns with UNESCO's SDG 4 monitoring fram

Attachment Submitted filename: a rebuttel letter.docx

# Peer review: Decision Letter 1

**DOI:** 10.1371/journal.pone.0347744.r003

**Authors:** Ye Wei

19 Aug 2025

PONE-D-25-16301R1

Research on the Evaluation and Spatial Layout of High Quality Development of Higher Education in China

PLOS ONE

Dear Dr. Li,

Thank you for submitting your manuscript to PLOS ONE. After careful consideration, we feel that it has merit but does not fully meet PLOS ONE’s publication criteria as it currently stands. Therefore, we invite you to submit a revised version of the manuscript that addresses the points raised during the review process.

Please submit your revised manuscript by Oct 03 2025 11:59PM. If you will need more time than this to complete your revisions, please reply to this message or contact the journal office at plosone@plos.org. When you're ready to submit your revision, log on to https://www.editorialmanager.com/pone/ and select the 'Submissions Needing Revision' folder to locate your manuscript file.

Please include the following items when submitting your revised manuscript:

- A rebuttal letter that responds to each point raised by the academic editor and reviewer(s). You should upload this letter as a separate file labeled 'Response to Reviewers'.
- A marked-up copy of your manuscript that highlights changes made to the original version. You should upload this as a separate file labeled 'Revised Manuscript with Track Changes'.
- An unmarked version of your revised paper without tracked changes. You should upload this as a separate file labeled 'Manuscript'.

If you would like to make changes to your financial disclosure, please include your updated statement in your cover letter. Guidelines for resubmitting your figure files are available below the reviewer comments at the end of this letter.

If applicable, we recommend that you deposit your laboratory protocols in protocols.io to enhance the reproducibility of your results. Protocols.io assigns your protocol its own identifier (DOI) so that it can be cited independently in the future. For instructions see: https://journals.plos.org/plosone/s/submission-guidelines#loc-laboratory-protocols. Additionally, PLOS ONE offers an option for publishing peer-reviewed Lab Protocol articles, which describe protocols hosted on protocols.io. Read more information on sharing protocols at https://plos.org/protocols?utm_medium=editorial-email&utm_source=authorletters&utm_campaign=protocols.

We look forward to receiving your revised manuscript.

Kind regards,

Ye Wei, PhD

Academic Editor

PLOS ONE

Journal Requirements:

1. If the reviewer comments include a recommendation to cite specific previously published works, please review and evaluate these publications to determine whether they are relevant and should be cited. There is no requirement to cite these works unless the editor has indicated otherwise.

[Note: HTML markup is below. Please do not edit.]

Reviewers' comments:

Reviewer's Responses to Questions

**Comments to the Author**

1. If the authors have adequately addressed your comments raised in a previous round of review and you feel that this manuscript is now acceptable for publication, you may indicate that here to bypass the “Comments to the Author” section, enter your conflict of interest statement in the “Confidential to Editor” section, and submit your "Accept" recommendation.

Reviewer #1: All comments have been addressed

Reviewer #3: All comments have been addressed

**********

2. Is the manuscript technically sound, and do the data support the conclusions?

The manuscript must describe a technically sound piece of scientific research with data that supports the conclusions. Experiments must have been conducted rigorously, with appropriate controls, replication, and sample sizes. The conclusions must be drawn appropriately based on the data presented.

Reviewer #1: Yes

Reviewer #3: Partly

**********

3. Has the statistical analysis been performed appropriately and rigorously?

Reviewer #1: Yes

Reviewer #3: No

**********

4. Have the authors made all data underlying the findings in their manuscript fully available?

The PLOS Data policy requires authors to make all data underlying the findings described in their manuscript fully available without restriction, with rare exception (please refer to the Data Availability Statement in the manuscript PDF file). The data should be provided as part of the manuscript or its supporting information, or deposited to a public repository. For example, in addition to summary statistics, the data points behind means, medians and variance measures should be available. If there are restrictions on publicly sharing data—e.g. participant privacy or use of data from a third party—those must be specified.

Reviewer #1: Yes

Reviewer #3: Yes

**********

5. Is the manuscript presented in an intelligible fashion and written in standard English?

PLOS ONE does not copyedit accepted manuscripts, so the language in submitted articles must be clear, correct, and unambiguous. Any typographical or grammatical errors should be corrected at revision, so please note any specific errors here.

Reviewer #1: Yes

Reviewer #3: Yes

**********

6. Review Comments to the Author

Please use the space provided to explain your answers to the questions above. You may also include additional comments for the author, including concerns about dual publication, research ethics, or publication ethics. (Please upload your review as an attachment if it exceeds 20,000 characters)

Reviewer #1: (No Response)

Reviewer #3: This paper tries to construct an indicator system for harmonious development of China’s higher education, and use the method of “single indicator quantification-multi indicator synthesis-multi criteria integration” (SMI-P) to determine the harmonious development index of China’s higher education.

Authors should provide a wide and complete overview. They could include the link among internationalization, learning organization, competitive edge, research and development. So, they could mention the following related topics:

* How research and development provide businesses a competitive edge.

* Efforts to innovate favouring the absorption of foreign direct investment so as to achieve beneficial outcomes from innovation.

* Synthetic indicators of quality of subjective life in rural and urban areas.

The hypotheses could appear as a result of literature review.

The data, model and methodology should be explained and justified based on the literature review. It should be justified whether the sample size is enough to apply the methodology and be able to interpret the results.

I wish the authors the best of success.

**********

7. PLOS authors have the option to publish the peer review history of their article (what does this mean?). If published, this will include your full peer review and any attached files.

If you choose “no”, your identity will remain anonymous but your review may still be made public.

**Do you want your identity to be public for this peer review?**  For information about this choice, including consent withdrawal, please see our Privacy Policy.

Reviewer #1: No

Reviewer #3: No

**********

[NOTE: If reviewer comments were submitted as an attachment file, they will be attached to this email and accessible via the submission site. Please log into your account, locate the manuscript record, and check for the action link "View Attachments". If this link does not appear, there are no attachment files.]

While revising your submission, please upload your figure files to the Preflight Analysis and Conversion Engine (PACE) digital diagnostic tool, https://pacev2.apexcovantage.com/. PACE helps ensure that figures meet PLOS requirements. To use PACE, you must first register as a user. Registration is free. Then, login and navigate to the UPLOAD tab, where you will find detailed instructions on how to use the tool. If you encounter any issues or have any questions when using PACE, please email PLOS at figures@plos.org. Please note that Supporting Information files do not need this step.

# Author comment: Author response to Decision Letter 2

**DOI:** 10.1371/journal.pone.0347744.r004

13 Sep 2025

Dear Editor and Reviewers:

We appreciate your positive and constructive comments, suggestions and language corrections on our manuscript entitled “Research on the Evaluation and Spatial Layout of High Quality Development of Higher Education in China (PONE-D-25-16301)”.

Reviewer 3:

This paper tries to construct an indicator system for harmonious development of China’s higher education, and use the method of “single indicator quantification-multi indicator synthesis-multi criteria integration” (SMI-P) to determine the harmonious development index of China’s higher education.

Authors should provide a wide and complete overview. They could include the link among internationalization, learning organization, competitive edge, research and development. So, they could mention the following related topics:

* How research and development provide businesses a competitive edge.

* Efforts to innovate favouring the absorption of foreign direct investment so as to achieve beneficial outcomes from innovation.

* Synthetic indicators of quality of subjective life in rural and urban areas.

The hypotheses could appear as a result of literature review.

The data, model and methodology should be explained and justified based on the literature review. It should be justified whether the sample size is enough to apply the methodology and be able to interpret the results.

I wish the authors the best of success.

Responses:

Thank you for your suggestion, We have made the following modifications.

1. Expanding the Literature Review

“Authors should provide a wide and complete overview. They could include the link among internationalization, learning organization, competitive edge, research and development.”

We appreciate the suggestion to broaden the literature review. We will integrate discussions on how research and development (R&D) enhances competitive advantage, the role of innovation in attracting foreign direct investment (FDI), and synthetic indicators of quality of life in urban/rural areas.

Revised Text: “Strategic learning, as a dynamic capability rooted in Senge’s learning organization theory, not only fosters continuous adaptation but also underpins the internationalization of higher education. For instance, R&D activities in universities provide businesses with a competitive edge by bridging academia-industry gaps. Similarly, regional innovation efforts—such as those measured by synthetic quality-of-life indicators in urban/rural areas—attract FDI, which in turn amplifies the outcomes of educational innovation. These linkages highlight the multifaceted role of higher education in driving sustainable development.”

(Line 100, Page 6)

2. Clarifying Hypotheses

“The hypotheses could appear as a result of literature review.”

We will explicitly state hypotheses derived from the literature review to strengthen the theoretical foundation.

Revised Text: “Based on the literature review, we hypothesize that: H1: Regional ecological civilization positively correlates with HDHE due to its role in sustainable development. H2: Higher student-teacher ratios negatively impact HDHE by straining resource allocation. H3: Internationalization (e.g., FDI absorption) moderates the relationship between R&D investment and HDHE.”

(Line 153, Page 8)

3. Justifying Methodology and Sample Size

“The data, model and methodology should be explained and justified based on the literature review... whether the sample size is enough.”

We will add justifications for the SMI-P method, Tobit model, and sample size (31 provinces over 23 years) with references to similar studies.

Revised Text: “Our panel dataset (31 provinces × 23 years) aligns with established spatial econometric studies, ensuring robustness for the SMI-P method, which is validated for multi-criteria integration in education research. The Tobit model is chosen to address censored data limitations, as demonstrated in regional development studies.”

(Line 238, Page 13)

These revisions address the reviewer’s concerns while maintaining the paper’s original focus. Let me know if further adjustments are needed!

Attachment Submitted filename: a_rebuttel_letter_auresp_2.docx

# Peer review: Decision Letter 2

**DOI:** 10.1371/journal.pone.0347744.r005

**Authors:** Ye Wei

29 Sep 2025

PONE-D-25-16301R2

Research on the Evaluation and Spatial Layout of High Quality Development of Higher Education in China

PLOS ONE

Dear Dr. Li,

Thank you for submitting your manuscript to PLOS ONE. After careful consideration, we feel that it has merit but does not fully meet PLOS ONE’s publication criteria as it currently stands. Therefore, we invite you to submit a revised version of the manuscript that addresses the points raised during the review process.

Please submit your revised manuscript by Nov 13 2025 11:59PM. If you will need more time than this to complete your revisions, please reply to this message or contact the journal office at plosone@plos.org. When you're ready to submit your revision, log on to https://www.editorialmanager.com/pone/ and select the 'Submissions Needing Revision' folder to locate your manuscript file.

Please include the following items when submitting your revised manuscript:

- A rebuttal letter that responds to each point raised by the academic editor and reviewer(s). You should upload this letter as a separate file labeled 'Response to Reviewers'.
- A marked-up copy of your manuscript that highlights changes made to the original version. You should upload this as a separate file labeled 'Revised Manuscript with Track Changes'.
- An unmarked version of your revised paper without tracked changes. You should upload this as a separate file labeled 'Manuscript'.

If you would like to make changes to your financial disclosure, please include your updated statement in your cover letter. Guidelines for resubmitting your figure files are available below the reviewer comments at the end of this letter.

If applicable, we recommend that you deposit your laboratory protocols in protocols.io to enhance the reproducibility of your results. Protocols.io assigns your protocol its own identifier (DOI) so that it can be cited independently in the future. For instructions see: https://journals.plos.org/plosone/s/submission-guidelines#loc-laboratory-protocols. Additionally, PLOS ONE offers an option for publishing peer-reviewed Lab Protocol articles, which describe protocols hosted on protocols.io. Read more information on sharing protocols at https://plos.org/protocols?utm_medium=editorial-email&utm_source=authorletters&utm_campaign=protocols.

We look forward to receiving your revised manuscript.

Kind regards,

Ye Wei, PhD

Academic Editor

PLOS ONE

Journal Requirements:

If the reviewer comments include a recommendation to cite specific previously published works, please review and evaluate these publications to determine whether they are relevant and should be cited. There is no requirement to cite these works unless the editor has indicated otherwise.

[Note: HTML markup is below. Please do not edit.]

Reviewers' comments:

Reviewer's Responses to Questions

**Comments to the Author**

1. If the authors have adequately addressed your comments raised in a previous round of review and you feel that this manuscript is now acceptable for publication, you may indicate that here to bypass the “Comments to the Author” section, enter your conflict of interest statement in the “Confidential to Editor” section, and submit your "Accept" recommendation.

Reviewer #3: All comments have been addressed

**********

2. Is the manuscript technically sound, and do the data support the conclusions?

The manuscript must describe a technically sound piece of scientific research with data that supports the conclusions. Experiments must have been conducted rigorously, with appropriate controls, replication, and sample sizes. The conclusions must be drawn appropriately based on the data presented.

Reviewer #3: Yes

**********

3. Has the statistical analysis been performed appropriately and rigorously?

Reviewer #3: Yes

**********

4. Have the authors made all data underlying the findings in their manuscript fully available?

The PLOS Data policy requires authors to make all data underlying the findings described in their manuscript fully available without restriction, with rare exception (please refer to the Data Availability Statement in the manuscript PDF file). The data should be provided as part of the manuscript or its supporting information, or deposited to a public repository. For example, in addition to summary statistics, the data points behind means, medians and variance measures should be available. If there are restrictions on publicly sharing data—e.g. participant privacy or use of data from a third party—those must be specified.

Reviewer #3: Yes

**********

5. Is the manuscript presented in an intelligible fashion and written in standard English?

PLOS ONE does not copyedit accepted manuscripts, so the language in submitted articles must be clear, correct, and unambiguous. Any typographical or grammatical errors should be corrected at revision, so please note any specific errors here.

Reviewer #3: Yes

**********

6. Review Comments to the Author

Please use the space provided to explain your answers to the questions above. You may also include additional comments for the author, including concerns about dual publication, research ethics, or publication ethics. (Please upload your review as an attachment if it exceeds 20,000 characters)

Reviewer #3: This paper could be improved. Authors should provide a wide and complete overview. They could include the link among internationalization, learning organization, competitive edge, research and development. So, they could mention the following related topics in countries such as Spain, Germany, among others:

* How research and development provide businesses a competitive edge.

* Efforts to innovate favouring the absorption of foreign direct investment so as to achieve beneficial outcomes from innovation.

* Synthetic indicators of quality of subjective life in rural and urban areas.

I wish the authors the best.

**********

7. PLOS authors have the option to publish the peer review history of their article (what does this mean?). If published, this will include your full peer review and any attached files.

If you choose “no”, your identity will remain anonymous but your review may still be made public.

**Do you want your identity to be public for this peer review?**  For information about this choice, including consent withdrawal, please see our Privacy Policy.

Reviewer #3: No

**********

[NOTE: If reviewer comments were submitted as an attachment file, they will be attached to this email and accessible via the submission site. Please log into your account, locate the manuscript record, and check for the action link "View Attachments". If this link does not appear, there are no attachment files.]

While revising your submission, please upload your figure files to the Preflight Analysis and Conversion Engine (PACE) digital diagnostic tool, https://pacev2.apexcovantage.com/. PACE helps ensure that figures meet PLOS requirements. To use PACE, you must first register as a user. Registration is free. Then, login and navigate to the UPLOAD tab, where you will find detailed instructions on how to use the tool. If you encounter any issues or have any questions when using PACE, please email PLOS at figures@plos.org. Please note that Supporting Information files do not need this step.

# Author comment: Author response to Decision Letter 3

**DOI:** 10.1371/journal.pone.0347744.r006

30 Jan 2026

Dear Editorial Team,

Thank you for your detailed feedback on our manuscript. We have carefully addressed the two issues raised and are pleased to submit the revised version for your review. Below is a detailed explanation of the revisions made:

1. Correction of File Categorization

We have thoroughly checked the file submissions and confirmed that all relevant files previously misclassified under "Other" have been re-categorized correctly as "Supplementary Files" in the Editorial Manager system. Each supplementary file is labeled with a standardized naming convention (e.g., "S2_Data.xlsx" ) and matched to the corresponding file type (e.g., "content-permission-form") as required by PLOS ONE’s submission guidelines. After rebuilding the files, we verified that all supplementary files are properly integrated into the submission package without omissions.

2. Replacement and Modification of Base Maps with CC BY 4.0 Licensed Data

To comply with the CC BY 4.0 license requirements, we have replaced the original base maps (sourced from RESDC and NBSC) with administrative boundary data obtained from geoBoundaries (https://www.geoboundaries.org/), a globally recognized open-access database explicitly licensed under CC BY 4.0. This license permits unrestricted reuse, redistribution, and commercial use by third parties, fully aligning with PLOS ONE’s open science policies.

Key details of the revisions are as follows:

1.The new base maps adopt provincial-level administrative boundary data (ADM1 level) from geoBoundaries, with the spatial reference system projected to WGS84 (EPSG: 4326) for consistency with other geographic datasets in the study.

2.Adaptive modifications have been made to the base maps to enhance relevance and visualization: irrelevant peripheral regions were clipped to match the research scope, boundary line widths were adjusted for clarity, and administrative division labels were added at key locations. Notably, the core geometric accuracy of the original boundaries remains unchanged.

3.We have attached screenshots of the data download page (labeled as "S1_GeoBoundaries_Download_Screenshot.pdf") as supplementary files, which clearly show the CC BY 4.0 license annotation and dataset details from the geoBoundaries website.

4.Detailed attributions for the base maps have been included in the figure captions of the revised manuscript, specifying the data source, license type, and download link (e.g., "Administrative boundary data were downloaded from geoBoundaries (https://www.geoboundaries.org/) under the Creative Commons Attribution 4.0 International License (CC BY 4.0)").

All revisions have been completed in accordance with PLOS ONE’s submission guidelines. We have rechecked the entire manuscript and supplementary files to ensure compliance, and the revised DOC has been rebuilt and approved through the Editorial Manager system.

Thank you again for your guidance and patience. We look forward to your further review of our submission. Please feel free to contact us if you require any additional information or clarification.

Sincerely,

Wen Li

# Peer review: Decision Letter 3

**DOI:** 10.1371/journal.pone.0347744.r007

**Authors:** Ye Wei

25 Feb 2026

PONE-D-25-16301R3

Research on the Evaluation and Spatial Layout of High Quality Development of Higher Education in China

PLOS One

Dear Dr. Li,

Thank you for submitting your manuscript to PLOS ONE. After careful consideration, we feel that it has merit but does not fully meet PLOS ONE’s publication criteria as it currently stands. Therefore, we invite you to submit a revised version of the manuscript that addresses the points raised during the review process.

Please submit your revised manuscript by Apr 11 2026 11:59PM. If you will need more time than this to complete your revisions, please reply to this message or contact the journal office at plosone@plos.org. When you're ready to submit your revision, log on to https://www.editorialmanager.com/pone/ and select the 'Submissions Needing Revision' folder to locate your manuscript file.

Please include the following items when submitting your revised manuscript:

- A letter that responds to each point raised by the academic editor and reviewer(s). You should upload this letter as a separate file labeled 'Response to Reviewers'.
- A marked-up copy of your manuscript that highlights changes made to the original version. You should upload this as a separate file labeled 'Revised Manuscript with Track Changes'.
- An unmarked version of your revised paper without tracked changes. You should upload this as a separate file labeled 'Manuscript'.

If you would like to make changes to your financial disclosure, please include your updated statement in your cover letter. Guidelines for resubmitting your figure files are available below the reviewer comments at the end of this letter.

If applicable, we recommend that you deposit your laboratory protocols in protocols.io to enhance the reproducibility of your results. Protocols.io assigns your protocol its own identifier (DOI) so that it can be cited independently in the future. For instructions see: https://journals.plos.org/plosone/s/submission-guidelines#loc-laboratory-protocols. Additionally, PLOS ONE offers an option for publishing peer-reviewed Lab Protocol articles, which describe protocols hosted on protocols.io. Read more information on sharing protocols at https://plos.org/protocols?utm_medium=editorial-email&utm_source=authorletters&utm_campaign=protocols.

We look forward to receiving your revised manuscript.

Kind regards,

Ye Wei, PhD

Academic Editor

PLOS One

Journal Requirements:

If the reviewer comments include a recommendation to cite specific previously published works, please review and evaluate these publications to determine whether they are relevant and should be cited. There is no requirement to cite these works unless the editor has indicated otherwise.

[Note: HTML markup is below. Please do not edit.]

Reviewers' comments:

Reviewer's Responses to Questions

**Comments to the Author**

1. If the authors have adequately addressed your comments raised in a previous round of review and you feel that this manuscript is now acceptable for publication, you may indicate that here to bypass the “Comments to the Author” section, enter your conflict of interest statement in the “Confidential to Editor” section, and submit your "Accept" recommendation.

Reviewer #3: All comments have been addressed

**********

2. Is the manuscript technically sound, and do the data support the conclusions?

The manuscript must describe a technically sound piece of scientific research with data that supports the conclusions. Experiments must have been conducted rigorously, with appropriate controls, replication, and sample sizes. The conclusions must be drawn appropriately based on the data presented.

Reviewer #3: Yes

**********

3. Has the statistical analysis been performed appropriately and rigorously?

Reviewer #3: Yes

**********

4. Have the authors made all data underlying the findings in their manuscript fully available?

The PLOS Data policy requires authors to make all data underlying the findings described in their manuscript fully available without restriction, with rare exception (please refer to the Data Availability Statement in the manuscript PDF file). The data should be provided as part of the manuscript or its supporting information, or deposited to a public repository. For example, in addition to summary statistics, the data points behind means, medians and variance measures should be available. If there are restrictions on publicly sharing data—e.g. participant privacy or use of data from a third party—those must be specified.

Reviewer #3: Yes

**********

5. Is the manuscript presented in an intelligible fashion and written in standard English?

PLOS ONE does not copyedit accepted manuscripts, so the language in submitted articles must be clear, correct, and unambiguous. Any typographical or grammatical errors should be corrected at revision, so please note any specific errors here.

Reviewer #3: Yes

**********

6. Review Comments to the Author

Please use the space provided to explain your answers to the questions above. You may also include additional comments for the author, including concerns about dual publication, research ethics, or publication ethics. (Please upload your review as an attachment if it exceeds 20,000 characters)

Reviewer #3: Some improvements could be made. Authors should provide a wide and complete overview. They could include the link among internationalization, learning organization, competitive edge, research and development. So, they could mention the following related topics in geographical areas such as European Union, Spain, Germany, among others:

* How research and development provide businesses a competitive edge.

* Efforts to innovate favouring the absorption of foreign direct investment so as to achieve beneficial outcomes frominnovation.

* Synthetic indicators of quality of subjective life in rural and urban areas.

* Effect of shift work on health and job satisfaction.

I wish the authors the best.

**********

7. PLOS authors have the option to publish the peer review history of their article (what does this mean?). If published, this will include your full peer review and any attached files.

If you choose “no”, your identity will remain anonymous but your review may still be made public.

**Do you want your identity to be public for this peer review?**  For information about this choice, including consent withdrawal, please see our Privacy Policy.

Reviewer #3: No

**********

[NOTE: If reviewer comments were submitted as an attachment file, they will be attached to this email and accessible via the submission site. Please log into your account, locate the manuscript record, and check for the action link "View Attachments". If this link does not appear, there are no attachment files.]

To ensure your figures meet our technical requirements, please review our figure guidelines: https://journals.plos.org/plosone/s/figures

You may also use PLOS’s free figure tool, NAAS, to help you prepare publication quality figures: https://journals.plos.org/plosone/s/figures#loc-tools-for-figure-preparation.

NAAS will assess whether your figures meet our technical requirements by comparing each figure against our figure specifications.

# Author comment: Author response to Decision Letter 4

**DOI:** 10.1371/journal.pone.0347744.r008

10 Mar 2026

Response to Reviewer Comments

We greatly appreciate the reviewer for the constructive and insightful comments and suggestions, which have significantly improved the quality of this manuscript. We have revised the manuscript carefully and thoroughly according to your advice. The main revisions are as follows:

We have provided a wider and more complete overview of the research field.

We have added and clarified the link among internationalization, learning organization, competitive edge, and research and development (R&D).

We have included discussions related to typical regions and countries such as the European Union, Spain, Germany, etc.

We have added the following topics as suggested:

How research and development provide businesses a competitive edge;

Efforts to innovate favouring the absorption of foreign direct investment so as to achieve beneficial outcomes from innovation;

Synthetic indicators of quality of subjective life in rural and urban areas;

Effect of shift work on health and job satisfaction.

All the revisions have been marked in the manuscript. We hope the revised version can meet the requirements of the journal. We sincerely thank you for your valuable time and professional comments.

Attachment Submitted filename: Response to Reviewer Comments.doc

# Peer review: Decision Letter 4

**DOI:** 10.1371/journal.pone.0347744.r009

**Authors:** Ye Wei

24 Mar 2026

PONE-D-25-16301R4

Research on the Evaluation and Spatial Layout of High Quality Development of Higher Education in China

PLOS One

Dear Dr. Li,

Thank you for submitting your manuscript to PLOS ONE. After careful consideration, we feel that it has merit but does not fully meet PLOS ONE’s publication criteria as it currently stands. Therefore, we invite you to submit a revised version of the manuscript that addresses the points raised during the review process.

Please submit your revised manuscript by May 08 2026 11:59PM. If you will need more time than this to complete your revisions, please reply to this message or contact the journal office at plosone@plos.org. When you're ready to submit your revision, log on to https://www.editorialmanager.com/pone/ and select the 'Submissions Needing Revision' folder to locate your manuscript file.

Please include the following items when submitting your revised manuscript:

- A letter that responds to each point raised by the academic editor and reviewer(s). You should upload this letter as a separate file labeled 'Response to Reviewers'.
- A marked-up copy of your manuscript that highlights changes made to the original version. You should upload this as a separate file labeled 'Revised Manuscript with Track Changes'.
- An unmarked version of your revised paper without tracked changes. You should upload this as a separate file labeled 'Manuscript'.

If you would like to make changes to your financial disclosure, please include your updated statement in your cover letter. Guidelines for resubmitting your figure files are available below the reviewer comments at the end of this letter.

If applicable, we recommend that you deposit your laboratory protocols in protocols.io to enhance the reproducibility of your results. Protocols.io assigns your protocol its own identifier (DOI) so that it can be cited independently in the future. For instructions see: https://journals.plos.org/plosone/s/submission-guidelines#loc-laboratory-protocols. Additionally, PLOS ONE offers an option for publishing peer-reviewed Lab Protocol articles, which describe protocols hosted on protocols.io. Read more information on sharing protocols at https://plos.org/protocols?utm_medium=editorial-email&utm_source=authorletters&utm_campaign=protocols.

We look forward to receiving your revised manuscript.

Kind regards,

Ye Wei, PhD

Academic Editor

PLOS One

Journal Requirements:

If the reviewer comments include a recommendation to cite specific previously published works, please review and evaluate these publications to determine whether they are relevant and should be cited. There is no requirement to cite these works unless the editor has indicated otherwise.

[Note: HTML markup is below. Please do not edit.]

Reviewers' comments:

Reviewer's Responses to Questions

**Comments to the Author**

1. If the authors have adequately addressed your comments raised in a previous round of review and you feel that this manuscript is now acceptable for publication, you may indicate that here to bypass the “Comments to the Author” section, enter your conflict of interest statement in the “Confidential to Editor” section, and submit your "Accept" recommendation.

Reviewer #3: All comments have been addressed

**********

2. Is the manuscript technically sound, and do the data support the conclusions?

The manuscript must describe a technically sound piece of scientific research with data that supports the conclusions. Experiments must have been conducted rigorously, with appropriate controls, replication, and sample sizes. The conclusions must be drawn appropriately based on the data presented.

Reviewer #3: Partly

**********

3. Has the statistical analysis been performed appropriately and rigorously?

Reviewer #3: Yes

**********

4. Have the authors made all data underlying the findings in their manuscript fully available?

The PLOS Data policy requires authors to make all data underlying the findings described in their manuscript fully available without restriction, with rare exception (please refer to the Data Availability Statement in the manuscript PDF file). The data should be provided as part of the manuscript or its supporting information, or deposited to a public repository. For example, in addition to summary statistics, the data points behind means, medians and variance measures should be available. If there are restrictions on publicly sharing data—e.g. participant privacy or use of data from a third party—those must be specified.

Reviewer #3: Yes

**********

5. Is the manuscript presented in an intelligible fashion and written in standard English?

PLOS ONE does not copyedit accepted manuscripts, so the language in submitted articles must be clear, correct, and unambiguous. Any typographical or grammatical errors should be corrected at revision, so please note any specific errors here.

Reviewer #3: Yes

**********

6. Review Comments to the Author

Please use the space provided to explain your answers to the questions above. You may also include additional comments for the author, including concerns about dual publication, research ethics, or publication ethics. (Please upload your review as an attachment if it exceeds 20,000 characters)

Reviewer #3: Some improvements could be made. Authors should provide a wide and complete overview in journals indexed in Journal Citation Report. They could include the link among internationalization, learning organization, competitive edge, research and development. So, they could mention the following related topics in geographical areas such as European Union, Spain, Germany, among others:

* How research and development provide businesses a competitive edge.

* Efforts to innovate favouring the absorption of foreign direct investment so as to achieve beneficial outcomes from innovation.

* Synthetic indicators of quality of subjective life in rural and urban areas.

* Effect of shift work on health and job satisfaction.

I wish the authors the best.

**********

7. PLOS authors have the option to publish the peer review history of their article (what does this mean?). If published, this will include your full peer review and any attached files.

If you choose “no”, your identity will remain anonymous but your review may still be made public.

**Do you want your identity to be public for this peer review?**  For information about this choice, including consent withdrawal, please see our Privacy Policy.

Reviewer #3: No

**********

[NOTE: If reviewer comments were submitted as an attachment file, they will be attached to this email and accessible via the submission site. Please log into your account, locate the manuscript record, and check for the action link "View Attachments". If this link does not appear, there are no attachment files.]

To ensure your figures meet our technical requirements, please review our figure guidelines: https://journals.plos.org/plosone/s/figures

You may also use PLOS’s free figure tool, NAAS, to help you prepare publication quality figures: https://journals.plos.org/plosone/s/figures#loc-tools-for-figure-preparation.

NAAS will assess whether your figures meet our technical requirements by comparing each figure against our figure specifications.

# Author comment: Author response to Decision Letter 5

**DOI:** 10.1371/journal.pone.0347744.r010

25 Mar 2026

Dear Reviewer�

We are deeply grateful for your professional, rigorous, and constructive comments on our manuscript entitled “Research on the Evaluation and Spatial Layout of High Quality Development of Higher Education in China (PONE-D-25-16301)”. We have carefully revised the manuscript in full accordance with your suggestions, and all revisions are clearly marked with tracks in the revised version for your easy checking.

Our point to point responses are as follows:

Comment 1

Some improvements could be made. Authors should provide a wide and complete overview in journals indexed in Journal Citation Report.

Response

We have supplemented and strengthened the comprehensive and systematic literature overview to meet the academic standards and narrative requirements of JCR indexed journals.

We expanded the theoretical framework connecting higher education spatial layout, economic society, and technological culture.

We clarified the research gaps and academic contributions more explicitly.

We strengthened the logical chain and international perspective to enhance readability and academic completeness for international readers.

Revision location: Section 1 Introduction

Comment 2

They could include the link among internationalization, learning organization, competitive edge, research and development.

Response

We have clearly established the internal logical link among internationalization, learning organization, competitive edge, and R&D.We added a clear narrative:Internationalization promotes cross border academic cooperation; learning organization enhances continuous innovation capability; R&D in universities helps enterprises form core competitiveness; and competitiveness further drives the high quality development of higher education.

Revision location: Section 1 Introduction

Comment 3

So, they could mention the following related topics in geographical areas such as European Union, Spain, Germany, among others:

1) How research and development provide businesses a competitive edge.

Response

We added content explaining that university R&D input and innovation output directly enhance industrial technology level and help enterprises obtain sustainable competitive advantage, and compared it with typical innovation practices in the European Union, Spain, and Germany.

Revision location: Section 3.1.1 The Temporal Changes of HDHE

2) Efforts to innovate favouring the absorption of foreign direct investment so as to achieve beneficial outcomes from innovation.

Response

We supplemented the view that regional innovation capacity helps attract FDI, and FDI further promotes knowledge spillover and technology transfer to amplify innovation benefits, and noted that this model has been widely used in the European Union, Spain, Germany, and other regions.

Revision location: Section 1 Introduction

3) Synthetic indicators of quality of subjective life in rural and urban areas.

Response

We pointed out that the “culture and life” module in our index system includes synthetic indicators reflecting subjective life quality in urban and rural areas, which is consistent with the indicator systems used in the European Union, Spain, and Germany.

Revision location: Section 2.3.1 Index System for Harmonious Development of Higher Education

4) Effect of shift work on health and job satisfaction.

Response

We added this topic as an important direction for future research, suggesting that future research can be extended to occupational health, exploring the effect of shift work on health and job satisfaction, as well as its relationship with higher education, talent quality, and well being.

Revision location: Section 4.2 Limitations and Future Research

Comment 4

I wish the authors the best.

Response

Thank you sincerely for your kindness and valuable guidance! We have completed all revisions earnestly and hope the revised manuscript can meet the standards of the journal.

Best regards,

Corresponding Author: Wen Li

full contact details:

Correspondence to Wen Li:

Email: liwen@ncwu.edu.cn

Date: 2026-03-25

place of employment: A teacher in North China University of Water Resources and Electric Power in China, Ph. D

postal address: North China University of Water Resources and Electric Power, No.136, Jinshui East Road, Zhengzhou 450046, P. R. China

Attachment Submitted filename: Response to Reviewer Comments.docx

# Peer review: Decision Letter 5

**DOI:** 10.1371/journal.pone.0347744.r011

**Authors:** Ye Wei

8 Apr 2026

Research on the Evaluation and Spatial Layout of High Quality Development of Higher Education in China

PONE-D-25-16301R5

Dear Dr. Li,

We’re pleased to inform you that your manuscript has been judged scientifically suitable for publication and will be formally accepted for publication once it meets all outstanding technical requirements.

Within one week, you’ll receive an e-mail detailing the required amendments. When these have been addressed, you’ll receive a formal acceptance letter and your manuscript will be scheduled for publication.

An invoice will be generated when your article is formally accepted. Please note, if your institution has a publishing partnership with PLOS and your article meets the relevant criteria, all or part of your publication costs will be covered. Please make sure your user information is up-to-date by logging into Editorial Manager at Editorial Manager® and clicking the ‘Update My Information' link at the top of the page. For questions related to billing, please contact billing support.

If your institution or institutions have a press office, please notify them about your upcoming paper to help maximize its impact. If they’ll be preparing press materials, please inform our press team as soon as possible -- no later than 48 hours after receiving the formal acceptance. Your manuscript will remain under strict press embargo until 2 pm Eastern Time on the date of publication. For more information, please contact onepress@plos.org.

Kind regards,

Ye Wei, PhD

Academic Editor

PLOS One

Additional Editor Comments (optional):

Reviewers' comments:

Reviewer's Responses to Questions

**Comments to the Author**

1. If the authors have adequately addressed your comments raised in a previous round of review and you feel that this manuscript is now acceptable for publication, you may indicate that here to bypass the “Comments to the Author” section, enter your conflict of interest statement in the “Confidential to Editor” section, and submit your "Accept" recommendation.

Reviewer #3: All comments have been addressed

**********

2. Is the manuscript technically sound, and do the data support the conclusions?

The manuscript must describe a technically sound piece of scientific research with data that supports the conclusions. Experiments must have been conducted rigorously, with appropriate controls, replication, and sample sizes. The conclusions must be drawn appropriately based on the data presented.

Reviewer #3: Partly

**********

3. Has the statistical analysis been performed appropriately and rigorously?

Reviewer #3: Yes

**********

4. Have the authors made all data underlying the findings in their manuscript fully available?

The PLOS Data policy requires authors to make all data underlying the findings described in their manuscript fully available without restriction, with rare exception (please refer to the Data Availability Statement in the manuscript PDF file). The data should be provided as part of the manuscript or its supporting information, or deposited to a public repository. For example, in addition to summary statistics, the data points behind means, medians and variance measures should be available. If there are restrictions on publicly sharing data—e.g. participant privacy or use of data from a third party—those must be specified.

Reviewer #3: Yes

**********

5. Is the manuscript presented in an intelligible fashion and written in standard English?

PLOS ONE does not copyedit accepted manuscripts, so the language in submitted articles must be clear, correct, and unambiguous. Any typographical or grammatical errors should be corrected at revision, so please note any specific errors here.

Reviewer #3: Yes

**********

6. Review Comments to the Author

Please use the space provided to explain your answers to the questions above. You may also include additional comments for the author, including concerns about dual publication, research ethics, or publication ethics. (Please upload your review as an attachment if it exceeds 20,000 characters)

Reviewer #3: I would like to commend the authors for their continued efforts in improving the manuscript throughout the review process. The paper addresses a relevant topic and shows clear progress.

Nevertheless, some improvements are still recommended. The literature review could be further strengthened by incorporating a broader and more comprehensive overview of recent international research, particularly from journals indexed in the Journal Citation Reports (JCR). In addition, the manuscript would benefit from a clearer articulation of the relationships among key concepts such as internationalization, learning organizations, competitive edge, and research and development. The authors may also consider integrating complementary perspectives from geographical contexts (e.g., the European Union, Spain, Germany), especially regarding:

How research and development provide businesses a competitive edge.

Efforts to innovate favouring the absorption of foreign direct investment so as to achieve beneficial outcomes from innovation.

Synthetic indicators of quality of subjective life in rural and urban areas.

Digital transformation and firm performance.

Overall, the manuscript has potential, and with these refinements, it could offer a more robust contribution to the field.

I wish the authors the best in their revisions.

**********

7. PLOS authors have the option to publish the peer review history of their article (what does this mean?). If published, this will include your full peer review and any attached files.

If you choose “no”, your identity will remain anonymous but your review may still be made public.

**Do you want your identity to be public for this peer review?**  For information about this choice, including consent withdrawal, please see our Privacy Policy.

Reviewer #3: No

**********

# Editor report: Acceptance letter

**DOI:** 10.1371/journal.pone.0347744.r012

**Authors:** Ye Wei

PONE-D-25-16301R5

PLOS One

Dear Dr. Li,

I'm pleased to inform you that your manuscript has been deemed suitable for publication in PLOS One. Congratulations! Your manuscript is now being handed over to our production team.

At this stage, our production department will prepare your paper for publication. This includes ensuring the following:

* All references, tables, and figures are properly cited

* All relevant supporting information is included in the manuscript submission,

* There are no issues that prevent the paper from being properly typeset

You will receive further instructions from the production team, including instructions on how to review your proof when it is ready. Please keep in mind that we are working through a large volume of accepted articles, so please give us a few days to review your paper and let you know the next and final steps.

Lastly, if your institution or institutions have a press office, please let them know about your upcoming paper now to help maximize its impact. If they'll be preparing press materials, please inform our press team within the next 48 hours. Your manuscript will remain under strict press embargo until 2 pm Eastern Time on the date of publication. For more information, please contact onepress@plos.org.

You will receive an invoice from PLOS for your publication fee after your manuscript has reached the completed accept phase. If you receive an email requesting payment before acceptance or for any other service, this may be a phishing scheme. Learn how to identify phishing emails and protect your accounts at https://explore.plos.org/phishing.

If we can help with anything else, please email us at customercare@plos.org.

Thank you for submitting your work to PLOS ONE and supporting open access.

Kind regards,

PLOS ONE Editorial Office Staff

on behalf of

Dr. Ye Wei

Academic Editor

PLOS One
